# Targeting PIP5K ameliorates hepatic cancer by inhibiting PI3K/AKT and the autophagy mechanism and enhancing ROS-mediated apoptosis

**DOI:** 10.3389/fphar.2026.1791462

**Published:** 2026-05-08

**Authors:** P. A. Shantanu, Bishal Rajdev, N. P. Syamprasad, Jagadeesh Kumar Gangasani, Arijit Mandal, Samir Ranjan Panda, Ramakrishna Sistla, Sai Balaji Andugulapati, Shiv Kumar Sarin, Dinesh Mani Tripathi, V. G. M. Naidu

**Affiliations:** 1 Department of Pharmacology & Toxicology, National Institute of Pharmaceutical Education and Research Guwahati, Guwahati, Assam, India; 2 Medicinal Chemistry and Pharmacology Division, CSIR-Indian Institute of Chemical Technology (IICT), Hyderabad, Telangana, India; 3 Liver Physiology and Vascular Biology Lab, Department of Molecular and Cellular Medicine, Institute of Liver and Biliary Sciences (ILBS), New Delhi, India

**Keywords:** autophagy, hepatocellular carcinoma, Nrf2, phosphatidylinositol-4-phosphate-5-kinase, proliferation, reactive oxygen species

## Abstract

**Introduction:**

Hepatic cancer cells regulate reactive oxygen species (ROS) and lipid kinases to promote proliferation and survival. The role of phosphatidylinositol-4-phosphate-5-kinase (PIP5K) in modulating the autophagy–Nrf2 antioxidant pathway remains unclear. In this study, we investigated the impact of PIP5K on the ROS-dependent autophagy–Nrf2 axis using specific isoform inhibitors (PIP5K1A: ISA-201IB; PIP5K1B: IITZ01) and further identified NG-TZ-17 and NG-TZ-20, which are analogs of IITZ01, as novel inhibitors of the PIP5KB isoform.

**Methods:**

The association between PIP5K and the ROS–autophagy–Nrf2 pathway was examined in hepatocellular carcinoma (HCC) tissue samples (n = 36) and hepatic cancer cell lines. *In vitro*, HepG2 cells (expressing high PIP5K levels) were treated with PIP5K isoform-specific inhibitors, autophagy inhibitors, and Nrf2 inhibitors in the presence of hydrogen peroxide (H_2_O_2_). The effects on ROS generation, proliferation, autophagy, antioxidant defense, and apoptosis were assessed using MitoSOX staining, immunofluorescence, and Western blot analysis. *In vivo*, SCID mice xenografted with GFP-HepG2 cells were treated orally with PIP5K inhibitors (IITZ01 and NG-TZ-17; 50 mg/kg) or sorafenib (60 mg/kg). Tumor progression was monitored through animal imaging, survival analysis, tumor volume measurement, and Western blotting of excised tumors.

**Results:**

PIP5K isoforms, Beclin-1, and Nrf2 expression increased with advancing HCC grade. Autophagy induction upregulated PIP5K isoforms, Nrf2, HO-1, and SOD2, thereby protecting cells from hydrogen peroxide-induced apoptosis. PIP5K inhibition enhanced ROS-mediated apoptosis by suppressing proliferation, autophagy, and Nrf2 signaling. *In vivo*, NG-TZ-17 and IITZ01 significantly reduced tumor burden, with efficacy comparable to sorafenib (*p* < 0.001 vs. vehicle control).

**Conclusion:**

PIP5K isoforms promote hepatic cancer cell proliferation under oxidative stress conditions. Targeting PIP5K sensitizes cells to ROS-mediated apoptosis through modulation of the PI3K/AKT/mTOR and autophagy pathways, highlighting PIP5K as a promising therapeutic target in hepatocellular carcinoma.

## Graphical Abstract

**Figure:**
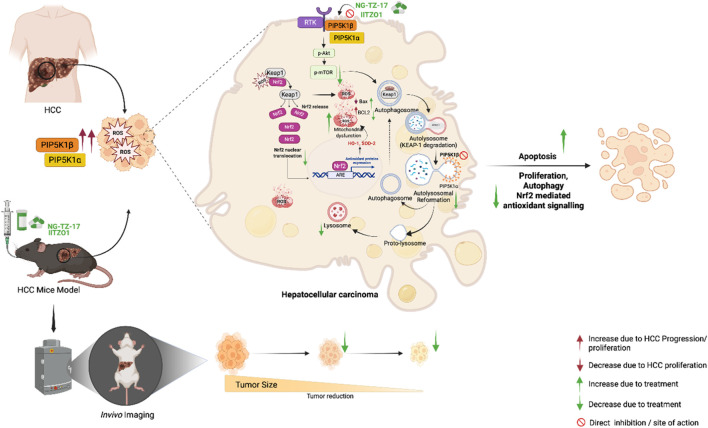

## 1 Introduction

Hepatocellular carcinoma (HCC) accounts for approximately 700,000 cancer deaths annually, and one million liver cancer cases are expected by 2025. Alcohol-associated liver disease (AALD), viral infections such as hepatitis B or C, aflatoxin B1 exposure, genetic mutations, and non-alcoholic fatty liver disease (NAFLD) are the major factors contributing to HCC (Llovet et al., 2021; Philips et al., 2021; Morgan et al., 2023). Impaired autophagy is a predominant mechanism associated with HCC arising from these diverse risk factors (Choi et al., 2018; Chao and Ding, 2019; Luo et al., 2021; Cao et al., 2022). Proliferative signals from various carcinogens overwhelm the evolutionarily conserved autophagy process, which is essential for cellular quality control. This impairment leads to the accumulation of oncogenic proteins, thereby driving the progression of carcinogenesis (Liu et al., 2017). Once a tumor is established, elevated autophagy promotes metabolic and adaptive rewiring to sustain tumor proliferation and manage the tumor microenvironment by restoring nutrient, oxygen, and energy supply (Liu et al., 2017; Shantanu et al., 2021).

Multiple studies have demonstrated a positive correlation between autophagy and proliferative markers during liver cancer progression, showing that phosphoinositide 3-kinase (PI3K) and autophagy pathways act together and reinforce each other (Smith and Macleod, 2019; Shantanu et al., 2021). High autophagy facilitates Nrf2 nuclear translocation and degrades Keap-1, thereby upregulating antioxidant enzymes such as SOD2 and HO-1. These adaptations allow cancer to grow under adverse conditions and promote resistance by countering ROS. Prolonged autophagy confers resistance to conventional and advanced chemotherapies, such as vascular endothelial growth factor (VEGF), mTOR, and PD-L1 inhibitors (Tang et al., 2016; Sun et al., 2017; Jiang et al., 2019; Smith and Macleod, 2019). Even the latest in-line PI3K/AKT inhibitors promote autophagy, which can lead to resistance and treatment failure (Lamoureux and Zoubeidi, 2013; Butler et al., 2017; Gremke et al., 2025).

Crucially, the cellular response to ROS follows a concentration-dependent response that dictates cellular fate (Ohguro et al., 1999). Mild ROS levels mimic the tumor microenvironment to drive proliferation via protective autophagy-mediated feedback, while moderate levels induce adaptive stasis, where nutrients are depleted, and high concentrations trigger a transition into autophagic cell death (Poillet-Perez et al., 2015; Redza-Dutordoir and Averill-Bates, 2021). Hence, identifying a common target involved in both proliferative and autophagy mechanisms may offer dual therapeutic benefit in cancer treatment.

Phosphatidylinositol-4-phosphate-5-kinase (PIP5K) is a target upstream of both the PI3K/AKT proliferative pathway and the autophagic-mediated adaptive pathway, especially in late autophagic events. PIP5K generates phosphatidylinositol-4,5-bisphosphate (PI4,5P_2_), which serves as a substrate for PI3K and recruits clathrin to enable lysosome recycling from autolysosomes during late autophagy. Thus, blocking PIP5K could potentially inhibit both proliferative and adaptive signaling (Van den Bout and Divecha, 2009; Moreau et al., 2012; Semenas et al., 2014; Chen et al., 2018; Chavez-Dominguez et al., 2020). Although the roles of PIP5K1A and PIP5K1C in HCC progression and metastasis are well established, no prior reports have examined the specific contribution of PIP5K1B to hepatic cancer progression (Peng et al., 2021).

In our initial work on the development of PI3K–mTOR dual inhibitors, we identified N2-[4-(1H-benzimidazol-2-yl)phenyl]-N4-(4-fluorophenyl)-6-(4-morpholinyl)-1,3,5-triazine-2,4-diamine (15g/IITZ01), which showed strong cytotoxic activity, despite only modest PI3K inhibition (Kumar et al., 2015). Follow-up studies revealed that treatment with this compound caused significant cytotoxicity, along with vacuolated cell morphology in breast cancer cells. These observations led us to examine the autophagy pathway, where we found that inhibition of autophagy was a key mechanism driving its activity (Guntuku et al., 2019). In this study, we investigated the role of PIP5K in hepatic cancer progression, optimized the lead compound IITZ01 to generate more potent derivatives, and examined the contribution of PIP5K isoforms to hepatic cancer cell survival using isoform-specific inhibitors in both *in vitro* and *in vivo* models. Importantly, these inhibitors not only delineated the mechanistic involvement of PIP5K but also demonstrated the potential to overcome the therapeutic limitations of the standard treatment, sorafenib (Ashokachakkaravarthy and Pottakkat, 2019).

## 2 Materials and methods

### 2.1 Materials

Tissue microarray slides were obtained from the DBT-National Liver Disease Biobank (NLDB) at the Institute of Liver and Biliary Sciences (ILBS), New Delhi, India. Immunohistochemistry was performed using the Vectastain® Universal Quick HRP Kit. Peroxidase (cat no. PK-8800) was purchased from Vector Technologies California, United States. The following cell lines were purchased from ATCC, Virginia, United States: PLC/PRF5 (cat no. CRL-8024), SNU-387 (cat no. CRL-2237), SK-Hep-1 (cat no. HTB-52), and HepG2 (cat no. HB-8065). Primary antibodies used for immunohistochemistry, immunofluorescence, and Western blot analysis, such as p-mTOR (cat no. 2972), alpha-tubulin (cat no. 2144), and Keap-1 primary antibody (cat no. D6B12); Phalloidin DyLight 554 (cat no. 13054); anti-rabbit HRP-conjugate secondary antibody (cat no. 7074S); and normal goat serum (cat no. 5425) were purchased from Cell Signaling Technologies (CST, Massachusetts, United States). Beclin-1 (cat no. ab207612) was purchased from Abcam, Cambridge, United Kingdom. PIP5K1A (cat no. A7941), PIP5K1B (cat no. A7749), p-AKT (cat no. A9272), SRC (cat no. A19119), SOD2 (cat no. A1346), HO-1 (cat no. A19062), ATG-5 (cat. A11427), AKR1B10 (cat no. A7823), and beta actin (cat no. AC026) were purchased from ABclonal, Massachusetts, United States. Additional reagents and kits were sourced as follows: ISA-2011B was sourced from Life technologies, California, United States. Hoechst 33258 (H1398), ProLong Gold Antifade (cat no. P36930) for mounting, Lipofectamine 2000 (cat no. 11668027), PI-4,5-P_2_ (cat no. ab11039), Premo™ Autophagy Tandem Sensor RFP-GFP-LC3B Kit (cat no. P36235), Annexin V/PI kit (cat no. BMS500FI-100), phosphatidylinositol 4,5-biphosphate (PIP_2_) monoclonal antibody (cat no. MA126438), goat anti-mouse secondary antibody Alexa Flour 647 (cat no. A21235), MitoSOX^TM^ (cat no. M36008), LysoTracker^TM^ Red-DND-99 (cat no. L7528), and acridine orange stains (cat no. A3568) were purchased from Thermo Fisher, Waltham, Massachusetts, United States. Ac-DEVD-AFC caspase-3 substrate (cat no. NC0664508) was purchased from Enzo Life Sciences, New York, United States. The pCDH-CMV-Nluc-P2A-copGFP-T2A-Puro plasmid (cat no. 73037) was purchased from Addgene, Massachusetts, United States. Hydrogen peroxide (cat no. H1009), N-acetyl-L-cysteine (NAC) (cat no. A7250), sorafenib (cat no. Y0002098), chloroquine (CQ, cat no. C6628), ML-385 (cat no. SML1833), extracellular matrix (cat no. E1270), RIPA buffer (cat no. R0278), and protease and phosphatase inhibitor cocktail (cat no. PPC2020) were purchased from Sigma Missouri, United States.

### 2.2 Expression levels of PIP5K and markers of autophagy in hepatic cancer

Tissue microarray slides, comprising the normal adjacent control and hepatic cancer specimens of grades 1–3, were obtained from the biobank facility at ILBS, New Delhi. Male and female patients with a histopathologically confirmed first-time diagnosis of HCC were retrospectively included. Tumors were graded using the Edmondson–Steiner system to classify patients into grades 1, 2, and 3. Uninvolved adjacent tissue (UAT) was collected at a distance of >2 cm from the tumor margin to ensure non-cancerous status. Immunohistochemical (IHC) scoring was performed using the IHC Profiler. Patient samples were excluded if they had received prior sorafenib or chemotherapy, exhibited secondary liver metastases, or had tissue cores lacking adequate histological grading. Additionally, cores with >50% necrosis or those with technical damage during tissue microarray (TMA) processing were excluded to maintain the accuracy of IHC and immunofluorescence (IF) measurements (Semenas et al., 2014; Peng et al., 2021). The slides were initially deparaffinized and rehydrated through sequential 3-min incubations with xylene and ethanol of decreasing concentrations. Antigen retrieval was performed by boiling the sections in citrate buffer (pH 6) for 20 min in a microwave, followed by washing with Tris Buffer Saline (TBS). The section antigens were then blocked with 5% BSA for 2 h. Subsequently, all slides were incubated with primary antibodies, followed by secondary antibodies conjugated with HRP-rabbit for IHC or anti-rabbit-Alexa-Fluor-488 for IF analysis. The slides were counterstained with hematoxylin (IHC) and Hoechst (IF) for nuclear staining. IHC slides were washed with 0.5% hydrogen peroxide (H_2_O_2_)–TBS and then incubated with DAB (chromogen). Both IF and IHC slides were washed, mounted, and viewed under a microscope. Bright-field and dark-field images of IHC and IF were captured using the EVOS Auto-2 microscope with ×20 and ×40 objective magnifications. The scoring of IHC was conducted using the IHC profiler in ImageJ software, and the fluorescent intensities of IF images were quantified using ImageJ software. The scores and fluorescent intensities of various hepatic cancer grades were then compared to those of normal adjacent control tissue sections.

### 2.3 Cell culture

All cells were cultured in minimum essential media (MEM) (Gibco, Life Technologies) supplemented with 10% FBS and 1% antibiotic solution, represented as complete media at 37 °C in a humidified CO_2_ incubator. Cell lines were authenticated by the distributor and were routinely tested for *mycoplasma* contamination. The basal expression of PIP5K isoforms, autophagy, and Nrf2 in various hepatic cancer cells was explored in cells growing in complete media.

### 2.4 Exploring the role of PIP5K in reactive oxygen species-mediated apoptosis and autophagy

Reactive oxygen species (ROS) are a common upstream marker controlling proliferation, autophagy, and apoptosis (Poillet-Perez et al.). According to the literature, treatment with 200 µM H_2_O_2_ inhibits PIP5K activity and is cytotoxic. The reported doubling time of HepG2 cells is approximately 48 h (Halstead et al., 2006; Omar et al., 2022). Therefore, to explore the effect of ROS on PIP5K, autophagy, and Nrf2, we treated HepG2 cells with increasing concentrations of H_2_O_2_ (6.25, 12.5, 25, 50, 100, and 200 μM) and explored its effect on 48-h cell viability (in the presence and absence of serum) using the MTT assay. The absorbance at 48 h was subtracted from the mean absorbance at 0 h to calculate proliferation. The subtracted absorbance of untreated cells was considered 100% to calculate the percent inhibition of proliferation. We further estimated the corresponding mitochondrial superoxide, lysosomal levels, and caspase-3 activity using MitoSOX, LysoTracker dyes, and a caspase-3 substrate assay, respectively, as HepG2 cells were treated with increasing concentrations of H_2_O_2_ in serum-starvation media for 24 h (Guntuku et al., 2019). To comprehend how PIP5K expression is altered in response to different ROS levels, molecular expression analysis by Western blotting was undertaken for the following markers: PIP5K1A, PIP5K1B, Beclin-1, Nrf2, Bax, and BCL-2. The changes observed were compared to those in cells growing in complete and starvation media.

### 2.5 Mitochondrial superoxide estimation through MitoSOX staining

HepG2 cells were treated serially with increasing concentrations of H_2_O_2_, as mentioned above, and in another set, cells were treated with NG-TZ-17 and IITZ01, along with standards ISA-2011B, CQ, and ML-385 for 24 h in the presence of mild (12.5 µM) H_2_O_2_. Cells were washed with PBS, followed by incubation with 1 μM MitoSOX stain in HBSS for 30 min. The stained cells were washed again, trypsinized, and centrifuged. The mean fluorescence was measured using an Attune-NxT flow cytometer. MitoSOX-stained H_2_O_2_-exposed cells treated with PIP5K and standard inhibitors were mounted in slides for visualization using an EVOS Auto-2 fluorescence microscope (396/610 nm) in ×40 objective magnification. The fluorescent intensities of MitoSOX-stained cells were quantified using ImageJ software.

### 2.6 Estimating the autophagic flux through LysoTracker staining

HepG2 cells were treated serially with increasing concentrations of H_2_O_2_, as mentioned above. After incubation, the cells were washed with PBS and incubated with 50 nM LysoTracker-red DND-99 stain in HBSS for 30 min. Thereafter, the cells were trypsinized and centrifuged, and the mean fluorescence was measured using an Attune-NxT flow cytometer in the case of H_2_O_2_-treated cells.

### 2.7 Estimating caspase-3 activity using the caspase substrate assay

The caspase-3 substrate assay was performed as described by Kuželová et al. (2007), with slight modifications. In brief, the treated and control cell lysates, prepared using protease–phosphatase inhibitor-free NP-40 lysis buffer, were incubated with the Ac-DEVD-AFC substrate diluted in HEPES buffer for 1 h in 37 °C in the dark. Fluorescence was then measured using a spectrofluorometric plate reader (FlexStation 3 Multi-mode microplate reader, Molecular Devices, United States), with excitation and emission wavelengths of 400 nm and 505 nm, respectively. Active caspase-3 was determined by the release of 7-amino-4-trifluoromethyl-coumarin (AFC) from the synthetic substrate Ac-DEVD-AFC (Enzo Life Sciences, cat no. NC0664508). Caspase-3 activity was normalized to the total protein content of the samples, and the data were expressed as the relative fold change in caspase-3 activity over the control.

### 2.8 Development of PIP5K inhibitors

Previously, we synthesized and evaluated the cytotoxicity of benzimidazole-containing s-triazine molecules; the identified compound IITZ01 (N2-[4-(1H-benzimidazol-2-yl)phenyl]-N4-(4-fluorophenyl)-6-(4-morpholinyl)-1,3,5-triazine-2,4-diamine) exhibited strong cytotoxicity with moderate PI3K inhibition (Kumar et al., 2015). The mechanistic evaluation showed that inhibition of autophagy underlies the cytotoxic nature of the molecule (Guntuku et al., 2019). Upon further investigation of the molecular target behind autophagy inhibition using a cell-free kinase inhibition assay, we observed that IITZ01 showed characteristic inhibition toward the PIP5K1B isoform. At a concentration of 1 µM, IITZ01 exhibited 8%, 66%, and 35% inhibition of PIP5K1A, PIP5K1B, and PIP5K1C isoforms, respectively (unpublished data). To optimize the lead molecule IITZ01, we synthesized and screened biologically active IITZ01 derivatives for PIP5K inhibition and assessed their effect on cell viability in HCC cell lines (such as PLC/PRF5, SNU-387, SK-Hep-1, and HepG2), autophagy inhibition (using acridine orange, GFP-RFP-LC3B-Premo autophagy sensor, and PIP5K–LysoTracker dual staining assays), the effect on PI-4,5-P_2_ levels (immunofluorescence assay), and induction of apoptosis using the Annexin V–PI assay.

### 2.9 Enzyme inhibition

The enzyme inhibition studies were outsourced to the Thermo Fisher Scientific SelectScreen^TM^ biochemical kinase profiling service using the Z-LYTE^TM^ screening protocol and assay conditions. Biologically active derivatives IITZ01, IITZ02, NG-TZ-17, and NG-TZ-20 (1 μM) were incubated with a reaction mix containing PI(3,4)P_2_ (substrate), ATP, the PIP5K1B enzyme, ADP, and ATP tracer antibodies for single-point enzyme inhibition. Subsequently, serial three-fold dilutions, starting from 1 μM across 10 concentration points, were tested for 1 h, followed by the addition of labeled anti-ADP-AlexaFluor^TM^ 647 antibody and EDTA to terminate the reaction. Fluorescence is measured, and IC_50_ values of active triazine derivatives were calculated from the concentration vs. percent inhibition graph.

### 2.10 Cell viability studies on PIP5K inhibitors in HCC cell lines

To estimate the IC_50_ of IITZ01 and its derivatives NG-TZ-17 and NG-TZ-20 in hepatic cancer cells, PRF5, SNU-387, SK-hep-1, and HepG2 were plated into 96-well plates. Once cells attained the desired morphology, cells were starved for 2 h and then treated with serially increasing concentrations (0.156, 0.312, 0.625, 1.25, 2.5, 5, 10, and 20 μM) of NG-TZ-17, IITZ01, ISA-2011B, and (1.25, 3.125, 6.25, 12.5, 25, 50, and 100 μM) CQ. Subsequently, cells were incubated for 48 h, followed by the addition of 10 μL of MTT (5 mg/mL) to each well and incubated for 4 h. Thereafter, the MTT-containing solution was replaced with DMSO to dissolve the formazan crystals, and the absorbance was measured at 570 nm. The percent cell viability was calculated by dividing the absorbance of treated cells by that of untreated cells.

### 2.11 Acridine orange staining

HepG2 cells were treated with triazine PIP5K inhibitors, followed by incubation with two concentrations of NG-TZ-17 (0.4 and 0.9 μM), IITZ01 (0.5 and 1 μM), and NG-TZ-20 (2.83 and 5.66 μM) for 24 h. Following the treatment period, cells were washed with PBS and stained with 1 μM acridine orange in plain media for 30 min, followed by PBS wash to remove excess stain. Furthermore, the cells were trypsinized, followed by centrifugation. The mean fluorescence intensities were measured, and shifts were analyzed using an Attune-NxT flow cytometer.

### 2.12 Establishing the phase-specific inhibition of autophagy upon triazine PIP5K inhibitor treatment through GFP-RFP-LC3B baculovirus transfection

HepG2 cells were cultured on coverslips and transfected with a baculovirus vector containing an acid-sensitive GFP and an acid-insensitive RFP-LC3B sequence (Premo™ Autophagy Tandem Sensor RFP-GFP-LC3B Kit), according to the manufacturer’s instructions. The transfected cells were starved by replacing complete media with plain media, followed by treatment with inhibitors for 24 h. The cells were fixed with 4% PFA for 20 min and mounted on slides with DAPI–gold antifade. The images were captured using a Leica confocal microscope with ×63 objective magnification. The GFP and RFP LC3B puncta were counted and plotted in a graph to indicate the shift in expression following treatment.

### 2.13 Effect of NG-TZ-17 treatment on PI-4,5-P_2_ levels

HepG2 cells were cultured in complete medium and subsequently switched to serum-starvation medium for treatment. Cells were exposed to NG-TZ-17 at concentrations of 62.5, 125, 250, 500, 1000, and 2000 nM for 24 h. Post-treatment, cells were washed, fixed with 4% paraformaldehyde, and permeabilized using 0.01% Triton X-100. Blocking was performed with 5% normal goat serum, followed by overnight incubation at 4 °C with a primary PI-4,5-P2 antibody (1:200). After washing, cells were incubated with Alexa Fluor 488-conjugated anti-rabbit secondary antibody (10 μg/mL) for 1.5 h. Nuclei and cytoskeletal structures were counterstained with Hoechst 3342 and Phalloidin-Red, respectively. Confocal images were acquired using a Leica microscope with ×63 magnification. Mean fluorescence intensities of PI-4,5-P2 were quantified, and the IC_50_ value for NG-TZ-17 was calculated.

### 2.14 Dual LysoTracker staining and PIP5K1B immunofluorescence

HepG2 cells were treated according to the treatment plan discussed previously and washed with PBSS following the treatment incubation period. The cells were incubated with 50 nM LysoTracker-red DND-99 stain in HBSS for 30 min. The excess stain was washed and fixed with 4% PFA. The fixed cells were permeabilized with 0.01% Triton-X, followed by blocking with 5% normal goat serum. Then, cells were incubated with the PIP5K1B antibody (1:200) overnight in a humidified chamber at 4 °C. The cells were then washed and incubated with a secondary anti-rabbit Alexa Fluor 488 (10 μg/mL)-conjugated antibody for 1.5 h. The cells were washed and mounted on slides with ProLong-DAPI–gold antifade solution. The slides were then visualized, and images were captured using a confocal microscope with ×63 magnification. The fluorescent intensities were measured in ImageJ.

### 2.15 Annexin-V–FITC PI staining

Fluorescence intensity of Annexin-V–FITC and PI-stained cells was measured using a spectrofluorometric plate reader (FlexStation 3 Multi-mode microplate reader, Molecular Devices, United States) with excitation at 488 nm and emission wavelengths at 530 nm for the Annexin V–FITC conjugate and 610 nm for PI. The percentage of the early apoptotic cell population was calculated comparing with the fluorescence of the FITC control, and the percentage of the late apoptotic cell population was calculated using the following formula: [(mean fluorescence intensity of experimental sample − mean fluorescence intensity of live cells)/(mean fluorescence intensity of heat-killed cells − mean fluorescence intensity of live cells)] ×100 (Chen et al., 2013).

### 2.16 Effect of PIP5K inhibition on ROS-mediated apoptosis

We also investigated the effect of newly synthesized IITZ01 derivatives on ROS-mediated apoptosis. Furthermore, to explore whether treatment with PIP5K inhibitors sensitized HepG2 cells to mild concentrations of H_2_O_2_ (12.5 µM), HepG2 cells were exposed to a mild concentration of H_2_O_2_ and treated with two concentrations [IC_25_ (L) and IC_50_ (H)] of investigational NG-TZ-17 (17) (0.5 and 1 μM), IITZ01 (0.5 and 1 μM), and NG-TZ-20 (20) (2.83 and 5.66 μM), along with single concentrations of standards 67.10 μM chloroquine (CQ, autophagy inhibitor), 7.76 μM ISA-2011B (PIP5K1A inhibitor), and 5 μM ML-385 (Nrf2 inhibitor), in the presence of a proliferative concentration of H_2_O_2_ for 24 h. Additionally, the effect of these compounds on mitochondrial superoxide was assessed using MitoSOX dye; autophagy turnover was assessed using LysoTracker and PIP5K1B dual staining, and caspase-3 activity was assessed as mentioned above.

### 2.17 Effect of PIP5K inhibition on autophagy and cellular proliferation

The impact on PIP5K1A, PIP5K1B, Beclin-1, Nrf2, Bax, and BCL-2 levels was assessed using a Western blot analysis. In the case of *in vitro* samples, after the treatment period, cells were lysed with a RIPA buffer-containing protease–phosphatase inhibitor cocktail, and protein concentration was estimated using the Bradford assay. An equal amount of protein was loaded for molecular weight-based separation using SDS–PAGE, followed by transferring it onto a nitrocellulose membrane for probing. The transferred nitrocellulose membrane was blocked using 5% BSA to prevent non-specific binding. The blocked nitrocellulose membrane was incubated with primary antibodies mTOR, Nrf2, PIP5K1A, PIP5K1B, Beclin-1, SRC, Beta-actin, SOD2, and HO-1 (1:1000) for 12 h, followed by incubation with a secondary antibody for 2 h. Furthermore, blots were developed using chemiluminescence [peroxide–luminol (ECL solution), Bio-Rad] and documented using the Fusion-FX Vilber Lourmat chemiluminescence system). The band’s relative quantification and analysis were performed using ImageJ software.

### 2.18 Exploring the effect of ROS clearance on PIP5K treatment

To validate our results, serum-starved HepG2 cells were first exposed to H_2_O_2_ (12.5 µM) and subsequently treated with the IC_50_ concentrations of NG-TZ-17 (0.94 µM), IITZ01 (1 µM), and ISA-2011B (7.76 µM) in the presence or absence of NAC (200 µM) for 24 h. The effects on oxidative stress, viability, and apoptosis were measured using the MitoSOX assay, MTT assay, and Annexin V–PI assay, respectively.

### 2.19 *In vivo* efficacy of PIP5K inhibitors in a HepG2 cell-induced tumor model

The animals were maintained in individually ventilated cages at 25 °C ± 5 °C, with a relative humidity of 50% ± 10% in a pathogen-free environment. All experiments were performed in accordance with the Institutional Animal Ethical Committee (IAEC-IICT-Hyderabad, India, approval number IICT/IAEC/012/2022). GFP-expressing HepG2 cells were developed as described earlier (Balaji et al., 2016; Narendar et al., 2025). In brief, HepG2 cells were transiently transfected with the pCDH-CMV-Nluc-P2A-copGFP-T2A-Puro construct (sourced from Addgene) using Lipofectamine 2000 (Invitrogen), following the manufacturer’s instructions. Forty-eight hours post-transfection, cells were subjected to repeated puromycin selection (1 μg/mL) across multiple passages until a stable and uniformly GFP-expressing cell population was established. GFP expression was verified by fluorescence microscopy. The HCC model was developed by dispersing 50 μL HepG2–GFP (1 × 10^7^) cells in a mixture of MEM and ECM gel matrix, followed by subcutaneous injection into 4–5-week-old SCID mice (Vivo Bio Tech Ltd., Telangana, India). Once tumors reached to 200 mm^3^, animals were randomly divided into different experimental groups (n = 4). NG-TZ-17 and IITZ01 were dissolved in a vehicle consisting of 5% DMSO + 10% PEG400 + 10% Tween 80 + 75% ddH_2_O, and standard sorafenib was dissolved in 30% captisol in water and administered every day, orally, for 10 days. NG-TZ-17 and IITZ01 were administered at 50 mg/kg, and standard sorafenib was administered at 60 mg/kg (Kuczynski et al., 2015; Guntuku et al., 2019). Moribund animals were sacrificed. Tumor volumes and animal weights were recorded by two independent observers blinded to the experimental groups. Tumor volumes were calculated using the formula TV = (l × b^2^)/2, where l is the tumor length and b is the tumor width. *In vivo* GFP intensities of tumors were recorded through the IVIS Spectrum *In Vivo* Imaging system (PerkinElmer, San Diego, CA, United States). After the experimental period, tumors were collected, weighed, and processed for exploring changes in protein expression by extracting proteins using tissue protein extraction reagent (T-PER) buffer, followed by Western blotting.

### 2.20 Statistical analysis

All data are presented as the mean ± SEM. The significance of variation between the groups was determined using the unpaired Student’s t-test or one-way analysis of variance (ANOVA), followed by Bonferroni’s multiple comparison tests using GraphPad Prism, version 8.0. A value of *p < 0.05 was considered statistically significant. All experiments were carried out in triplicate unless otherwise specified.

## 3 Results

### 3.1 PIP5K expression increased along with autophagy and antioxidant markers in HCC patient samples and cell lines

**FIGURE 1:**
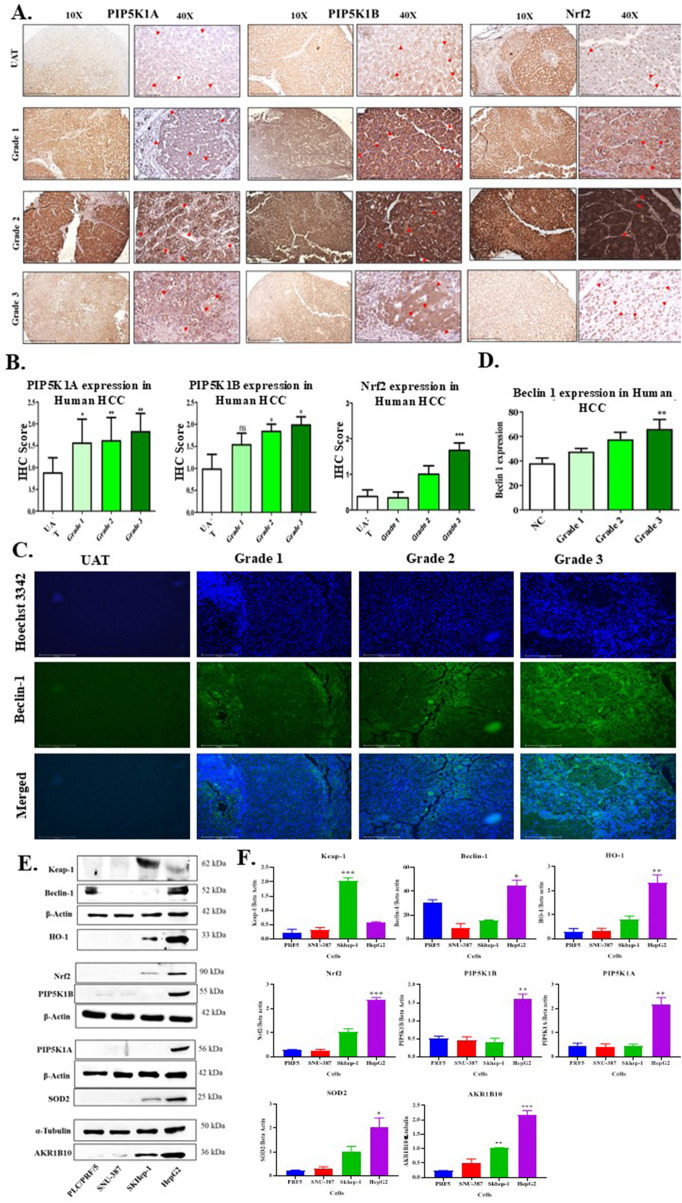
In hepatic cancer patient samples and cell lines, PIP5K expression increased in tandem with autophagy and antioxidant indicators. **(A)** IHC of Nrf2, PIP5K1A, and PIP5K1B. **(B)** IHC score graphs for Nrf2, PIP5K1A, and PIP5K1B 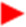 indicates positive cells. **(C)** Beclin-1 immunofluorescence. **(D)** Beclin-1 quantification. IHC and IF (n = 36). PIP5K isoforms, Nrf2, and autophagy marker (Beclin-1) expressions were overexpressed in the malignant tissue relative to adjacent tissue (AT), according to the IHC and immunofluorescence data, showing cells that are positive. IHC magnification: ×40 (scale bar: 100 μm) and ×10 (scale bar: 500 µm). Beclin-1 immunofluorescence pictures magnification: ×20 (scale bar: 125 μm) and ×10 (scale bar: 275 µm). **(E)** Western blots measuring the baseline levels of Beclin-1, Nrf2, SOD2, HO-1, AKR1B10, and PIP5K isoforms in PRF5, SNU-387, SK-Hep-1, and HepG2. **(F)** The bar graph depicts the quantification of Western blots (n = 3). Western blot results suggest a probable link between PIP5K–autophagy and antioxidant defense enzymes in hepatic cancer cell lines. Data were analyzed as the mean ± SEM using one-way ANOVA and the *post-hoc* Dunnett’s test. *, **, and *** indicate significant differences (*p* < 0.05, *p* < 0.01, and *p* < 0.001, respectively) compared to the AT or PLC/PRF5 cell lines.

To investigate the potential link between PIP5K, autophagy, and Nrf2 and explore the role of PIP5K in the progression of hepatic cancer, we estimated the expression of PIP5K1A, PIP5K1B, Beclin-1, and Nrf2 in 36 human HCC patient samples. We compared the expressions of these markers in various cancer grades (G1–G3) with normal adjacent control tissues (UATs). We observed a grade-dependent increase in the expressions of PIP5K1A, PIP5K1B, Beclin-1, and Nrf2. PIP5K1A and PIP5K1B expressions were elevated across all three cancer grades: G1, G3 (*p < 0.05), and G2 (**p < 0.01). We observed the highest expression of PIP5K isoforms in G2. A similar trend was observed in the expression of Nrf2 [G1 *(*p* < 0.05) and G2 **(*p* < 0.01)]. However, in the case of the autophagy marker Beclin-1, we observed a steady grade-dependent increase in expression, with the highest expression observed in the G3 grade (**, *p* < 0.01), compared to normal adjacent control tissues (Figures 1A–D).

A similar trend of positive correlation between PIP5K isoforms, Beclin-1, and Nrf2 was observed when we estimated the basal expression level in various HCC cell lines, such as PRF5, SNU387, SK-hep-1, and HepG2 (Figures 1E,F). The expression levels of PIP5K isoforms, Beclin-1, and Nrf2 were low in PRF5 and SNU-387 cell lines, whereas the expressions of PIP5K isoforms (PIP5K1A and B) were highest in HepG2 cells (**p < 0.01 for both PIP5K1A and B) compared to PRF5 cells. HepG2 also had higher levels of Beclin-1 and Nrf2 (*p < 0.05 for Beclin-1 and ***p < 0.001 for Nrf2 compared to PLC/PRF5). Keap-1 regulates Nrf2 nuclear translocation by forming a complex with Nrf2 and facilitating its degradation (Jiang et al., 2015). Basal Keap-1 levels were low in HepG2 and highest in SK-hep-1 (***p < 0.001 compared to PRF5). The Nrf2-dependent antioxidant enzymes such as HO-1 (**p < 0.01), SOD2 (*p < 0.05), and AKR1B10 (***p < 0.001) were also elevated in HepG2 compared to the PRF5 cell line (Figures 1C,D). Thus, since HepG2 expresses high basal levels of PIP5K isoforms, autophagy, and Nrf2-mediated antioxidant markers, HepG2 cells were selected for further experimentation.

### 3.2 PIP5K resists ROS-mediated autophagic cell death

**FIGURE 2:**
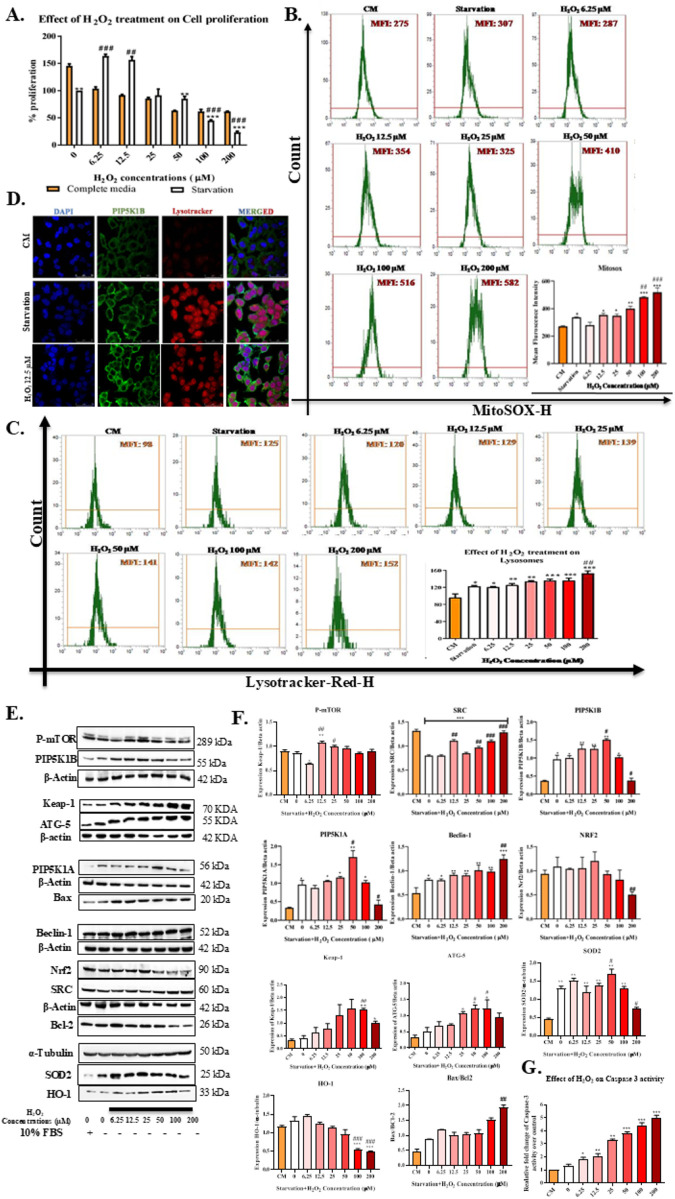
PIP5K resists ROS-mediated autophagic cell death. **(A)** Effect of ROS on cell viability: HepG2 cells were exposed to increasing concentrations of H_2_O_2_ (6.25–200 µM) with or without serum for 48 h. Proliferation inhibition (%) was calculated by normalizing the decrease in MTT absorbance post-treatment to baseline (pre-treatment) levels, considering the difference as 100% inhibition. In serum-free media, lower doses of H_2_O_2_ promote growth due to autophagy-mediated antioxidant pathways, leading to increased cell proliferation. At higher doses, ROS triggers autophagic cell death. In serum-containing media, this dose-dependent effect is not observed. To examine the impact of increasing H_2_O_2_ concentrations on ROS, autophagy flux, proliferation, antioxidant defense, and apoptosis, HepG2 cells were treated in serum-starved media for 24 h. Mitochondrial superoxide, lysosomal turnover, and PIP5K were assessed via staining, while pathway-specific markers were analyzed through Western blot. **(B)** Mitochondrial superoxide (MitoSOX) staining: H_2_O_2_ exposure increases mitochondrial superoxide under both media conditions. **(C)** Lysosome turnover (LysoTracker staining): H_2_O_2_ exposure increases lysosome turnover. **(D)** PIP5K1B and LysoTracker dual staining: Higher H_2_O_2_ levels increase ROS to cytotoxic levels, enhancing autophagy and leading to autophagic cell death. **(E)** Western Blots: **(F)** Quantification of Western blots. **(G)** Caspase 3 activity assay. PIP5K inhibited H_2_O_2_-induced cytotoxicity in HepG2 cells. Starvation and H_2_O_2_ induced PIP5K1A and B, SRC, Beclin-1, ATG 5, Nrf2, and antioxidant enzymes (HO-1 and SOD2), with a moderate increase in caspase-3 activity. At cytotoxic H_2_O_2_ levels (100 and 200 µM), these markers are downregulated, leading to oxidative stress-induced cell death indicated by an increase in the Bax/BCL-2 ratio and a substantial increase in caspase-3 activity. Data representation (n = 3): *, **, and *** represent *p* < 0.05, *p* < 0.01, and *p* < 0.001, respectively, when compared with the complete media control (CM), whereas #, ##, and ### represent *p* < 0.05, *p* < 0.01, and *p* < 0.001, respectively, when compared with the serum starvation control (starvation control). Data are represented as the mean ± SEM, followed by one-way ANOVA with Tukey’s *post-hoc* test (n = 3).

ROS governs various cell phases, such as proliferation, adaptation, and death by modulating the expression levels of lipid kinase (Poillet-Perez et al., 2015; Koundouros and Poulogiannis, 2018). Therefore, to establish the role of ROS in the modulation of PIP5K and autophagy–Nrf2 adaptive axis, we treated HepG2 cells with increasing concentrations of H_2_O_2_ (6.25, 12.5, 25, 50, 100, and 200 μM) and explored its effect on cell viability, mitochondrial superoxide (ROS), lysosome turnover (autophagy flux), expression of PI3K/AKT, autophagy, antioxidant, and apoptosis markers (Figure 2).

#### 3.2.1 Concentration-dependent effects of H_2_O_2_ concentrations

The effect on proliferation, stasis, and death to increasing concentrations of H_2_O_2_ was evaluated using the MTT assay. H_2_O_2_ at 6.25 μM (###, *p* < 0.001 vs. untreated cells) and 12.5 μM (##, *p* < 0.01 vs. untreated cells) promoted proliferation, while H_2_O_2_ at 25 and 50 μM cellular proliferation was reduced. However, exposure to 100 and 200 μM H_2_O_2_ resulted in significant cell death. Interestingly, serum-containing media did not show a concentration-dependent response to increasing H_2_O_2_ levels; instead, cell proliferation steadily decreased as the H_2_O_2_ concentration increased (Figure 2A).

#### 3.2.2 Effect on mitochondrial superoxides

The MitoSOX assay exhibited that mitochondrial superoxide concentration following H_2_O_2_ exposure correspondingly increased with H_2_O_2_ concentration. Serum starvation increases ROS compared to cells growing in complete media [*, *p* < 0.05 compared to complete media (CM)]. The ROS levels remained fairly unchanged even at increased H_2_O_2_ concentrations (50 μM). However, at cytotoxic concentrations of 100 and 200 μM, mitochondrial superoxide further increased compared to the starvation control. The increase in mitochondrial superoxide concentration was ## *p* < 0.01 and ### *p* < 0.001, respectively, compared to the serum-starvation control (Figure 2B).

#### 3.2.3 Effect on lysosomal turnover

Concurrently, when we measured lysosomal turnover indicative of autophagic flux, it increased following starvation (*, *p* < 0.05 compared to CM); following exposure to H_2_O_2_ in serum-starved cells, we observed a dose-dependent increase in lysosomal turnover compared to CM (Figure 2C). H_2_O_2_ at 200 μM induced autophagic lysosomal turnover more than serum starvation (###, *p* < 0.001 compared to serum starvation), resulting in autophagic cell death (Figure 2C). Taken together, the results of cellular proliferation, MitoSOX, LysoTracker staining, and mitochondrial superoxide (ROS) indicated that autophagy was activated. The elevated autophagy resisted an increase in mitochondrial superoxide at mild concentrations (6.25 and 12.5 μM), which induced cellular proliferation at moderate concentrations (25 and 50 μM) and promoted cellular stasis. However, the cytotoxic concentrations of 100 and 200 μM enhanced ROS to cytotoxic levels, thereby overactivating autophagy and leading to autophagic cell death (Figure 2C).

#### 3.2.4 Molecular changes following H_2_O_2_ treatment

Dual PIP5K1B and LysoTracker staining indicated that both PIP5K1B expression and lysosomal turnover are elevated at 12.5 μM H_2_O_2_ (Figure 2D). Furthermore, effects of exposure to increasing concentrations of H_2_O_2_ on PIP5K isoforms (PIP5K1A and PIP5K1B), Nrf2, SOD2, and HO-1 were evaluated through Western blotting. Compared to CM, serum starvation alone increased the expression of SOD2 (**, *p* < 0.01) and PIP5K isoforms (PIP5K1A and PIP5K1B) (*, *p* < 0.05). When H_2_O_2_ concentrations progressively increased to 50 μM, there was a proportional increase in the expression of PIP5K1A (**p < 0.001 vs. CM and #*p* < 0.05 vs. starvation), PIP5K1B (**p < 0.01 vs. CM and #*p* < 0.05 vs. starvation), Beclin-1 (**p < 0.01), ATG5 (*p < 0.05 vs. CM and #*p* < 0.05 vs. starvation), and SOD2 (**p < 0.01 vs. CM and #*p* < 0.05 vs. starvation) (Figures 2E,F). However, expressions of Nrf2, HO-1, Bax, and BCL-2 remained unaffected up to 50 μM H_2_O_2_. Nevertheless, PIP5K1A, PIP5K1B, Nrf2 (##, *p* < 0.01 vs. starvation), SOD2 (#, *p* < 0.01 vs. starvation), and HO-1 (###, *p* < 0.001 vs. starvation) expressions were downregulated at a cytotoxic concentration, particularly at 200 μM. ATG 5 (*p < 0.05 vs. CM and #*p* < 0.05 vs. starvation control) and Keap-1 (**p < 0.05 vs. CM and ##*p* < 0.05 vs. starvation control) expressions were induced as H_2_O_2_ concentration increased and was the highest at 100 µM. Compared to the serum-starvation control, SRC, a marker that moves PIP5K from membrane-based to cytosolic signaling, gradually increased and was the highest at 200 μM (##, *p* < 0.001 vs. starvation); a similar trend was observed in the case of Beclin-1 (***, *p* < 0.001 vs. CM and #, *p* < 0.01 vs. starvation). At 200 μM, Keap-1 expression was reduced but remained significantly higher than that in the starvation group (#*p* < 0.05 vs. starvation group). The Bax/BCL-2 ratio, indicative of apoptosis, increased after exposure to 200 μM H_2_O_2_ (##, *p* < 0.001 vs. starvation). Caspase-3 activity increased progressively and in a dose-dependent manner with increasing concentrations of H_2_O_2_, reaching its peak at the cytotoxic doses of 100 and 200 µM (***, *p* < 0.001 vs. CM and ###, *p* < 0.001 vs. starvation media) (Figure 2G). These results indicated that the cell adapts by increasing PIP5K expression to maintain both PI3K/AKT proliferative and autophagy signaling, upregulating Nrf2-mediated antioxidant defense markers; increased SRC indicates that a shift from membrane-based proliferative PI3K/AKT signaling to cytosolic autophagy signaling (Chen et al., 2009; Lowell, 2011). However, at a cytotoxic concentration of 200 μM, the expressions of PIP5K isoforms, Nrf2, SOD2, HO-1, and ATG5 decreased, while SRC and Beclin-1 increased. This indicates that at high concentration of H_2_O_2_ (ROS), cells could not modulate ROS even by upregulating autophagy and via the Nrf2-mediated antioxidant defense pathway, thus resulting in overactivation of autophagy and causing autophagic cell death (Figures 2E–G). Therefore, to mimic the tumor microenvironment characterized by elevated ROS and nutrient deprivation, serum-starved HepG2 cells were exposed to a mild concentration of 12.5 µM H_2_O_2_ as this condition consistently promoted proliferation and activated both autophagy and antioxidant defense markers, effectively representing the proliferative phase of the ROS gradient.

### 3.3 Identification of novel PIP5K inhibitors

**FIGURE 3:**
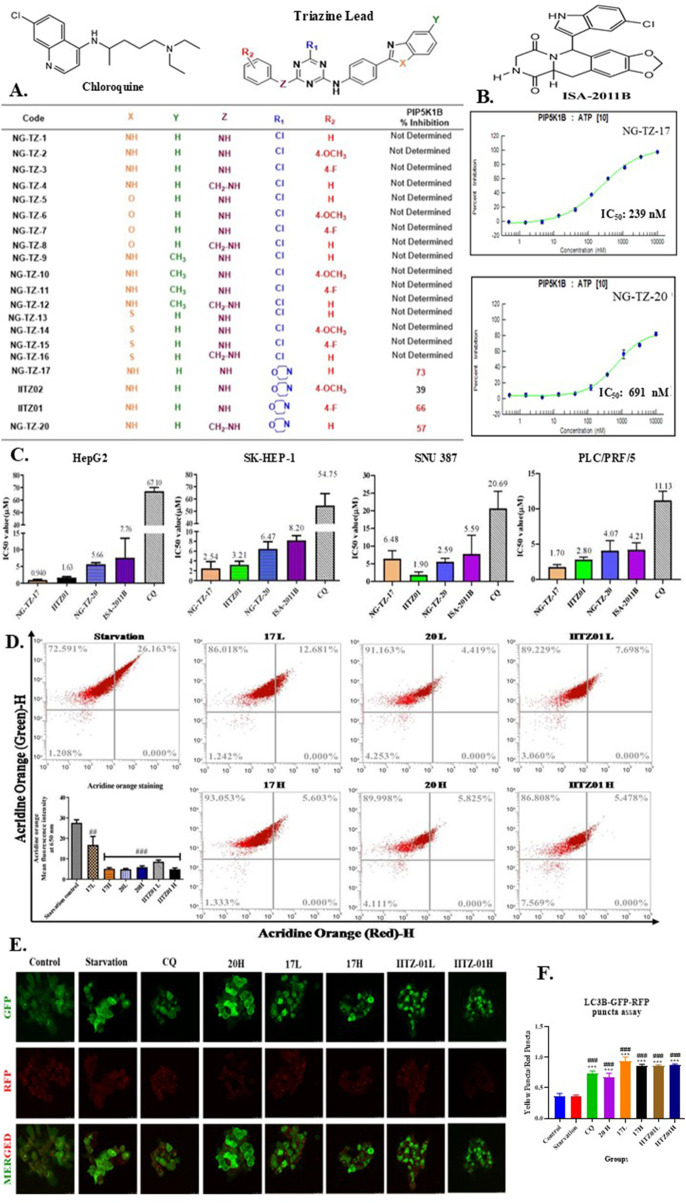
Triazine PIP5K inhibitors NG-TZ-17 and IITZ01 inhibited autophagy. **(A)** Table showing % PIP5K1B inhibition at 1 μM concentration. NG-TZ-17 and 20 were identified to exhibit 73% and 50% inhibition at 1 μM concentration. **(B)** The half-maximal inhibitory concentration (IC_50_) of lead compounds for PIP5K1B enzyme inhibition. IC_50_ values of NG-TZ-17 and 20 were 239 nM and 691 nM, respectively. **(C)** IC_50_ in various hepatic cancer cell lines (such as HepG2, SNU-387, SK-Hep-1, and PRF5). The triazine PIP5K inhibitors such as NG-TZ-17, IITZ01, and NG-TZ-20 were superior to standard PIP5K1A and autophagy inhibitors, such as ISA-2011B and chloroquine, in inhibiting cell proliferation. Data are represented as the mean ± SEM (n = 3). To evaluate the effects of NG-TZ-17, 20, and IITZ01 on autophagy, we treated HepG2 cells with IC_25_ (L) and IC_50_ (H) concentrations of these inhibitors for 24 h, followed by staining and LC3B-GFP-RFP autophagy sensor assays **(D)** Acridine orange red assay to measure the autophagic flux. Acridine orange is an acidotrophic dye that preferentially accumulates in acidic vesicles such as autolysosomes and lysosomes, and the treatment with PIP5K inhibitors resulted in a decrease in acridine orange fluorescence, indicating inhibition of the autophagic flux **(E)** Autophagy flux LC3A/B GFP-RFP puncta assay **(F)** Quantification of yellow puncta over red puncta. Successful baculovirus transfection results in the incorporation of LC3-GFP-RFP puncta (intracellular protein aggregates), which facilitates the fusion of lysosomes with autophagosomes during the autophagic flux; as PIP5K is involved in the recruitment of clathrin, inhibition of PIP5K will result in inhibition of PIP5K at the autolysosome step. The treatment with PIP5K inhibitors resulted in an increase in yellow/red puncta, indicative of inhibition of autophagy at the autolysosomal step. **(F)** Quantification of LC3A/B puncta. #, ##, and ### represent *p* < 0.05, *p* < 0.01, and *p* < 0.001, respectively, when compared with the serum starvation control (starvation control). Data are represented as the mean ± SEM, followed by one-way ANOVA with Tukey’s *post-hoc* test (n = 3).

Under lead optimization, biologically active derivatives of IITZ01 were synthesized, characterized, and further screened for PIP5K inhibition. The details of the synthesis and characterization of active triazine molecules are available in our previously published works (Jagadeesh Kumar et al., 2013; Kumar et al., 2015). Out of all the analogs synthesized, it was identified that NG-TZ-17 and NG-TZ-20 exhibited PIP5K1B inhibition of 73% and 57%, respectively, at 1 μM concentration (Figure 3A) and showed less than 50% inhibition on other isoforms. Hence, further dose–response curves for NG-TZ-17 and NG-TZ-20 were evaluated for dose-dependent inhibition of PIP5K1B, and the IC_50_ values were found to be 239 and 691 nM, respectively (Figure 3B). Structure–activity relationship analysis revealed that compounds with substitution on the phenyl group (IITZ01 and NG-TZ-20) showed decreased activity compared to the simple phenyl group (NG-TZ-17).

### 3.4 Comparative efficacy of triazine PIP5K inhibitors and standard inhibitors in hepatic cancer cell lines

We explored the efficacy of investigational triazine PIP5K inhibitors IITZ01, NG-TZ-17, and NG-TZ-20, along with standard inhibitors of PIP5K1A and autophagy, ISA-2011B and CQ, respectively, in hepatic cancer cell lines such as HepG2, SNU-387, SK-Hep-1, and PRF5 (Figure 3C). The IC_50_ values of NG-TZ-17, IITZ01, NG-TZ-20, ISA-2011B, and CQ in hepatic cancer are listed in Table 1.

**TABLE 1:** IC_50_ values (μM) of triazine PIP5K inhibitors and standards across the hepatic cancer cell line.

Cell line	NG-TZ-17	IITZ01	NG-TZ-20	ISA-2011B	Chloroquine (CQ)
HepG2	0.94	1.63	5.66	7.76	67.10
SNU-387	6.48	1.87	2.59	5.59	20.69
Skep-1	2.54	3.21	6.47	8.20	54.75
PRF5	1.70	2.80	4.07	4.21	11.13

**FIGURE 4:**
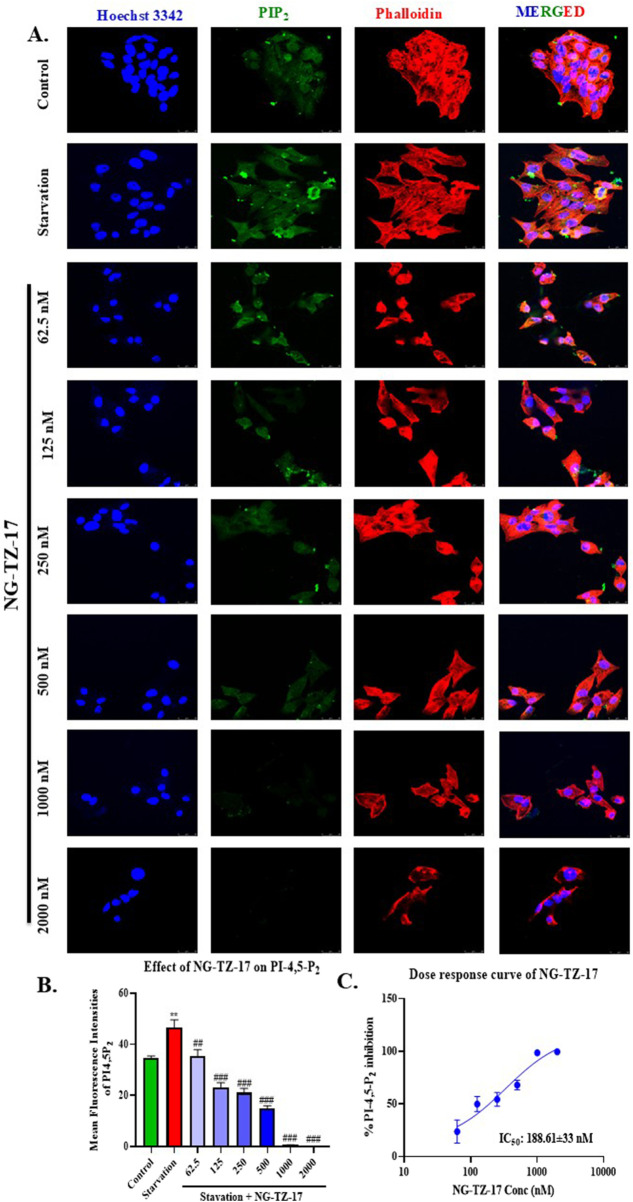
NG-TZ-17 treatment reduced PI-4,5-P2 levels in HepG2 cells **(A)** Representative confocal microscopy images of untreated and NG-TZ-17-treated HepG2 cells. **(B)** Quantitative analysis of mean fluorescence intensity of PI-4,5-P2. **(C)** Dose–response curve illustrating the effect of NG-TZ-17 on PI-4,5-P2 levels. HepG2 cells were cultured in complete medium. For treatment, the medium was replaced with serum-starvation medium, followed by exposure to increasing concentrations of NG-TZ-17 (62.5–2,000 nM) for 24 h. NG-TZ-17 treatment resulted in a significant reduction in PI-4,5-P2 levels. The half-maximal inhibitory concentration (IC_50_) of NG-TZ-17 for PI-4,5-P2 depletion in HepG2 cells was determined to be 188 ± 33 nM (n = 3).

**FIGURE 5:**
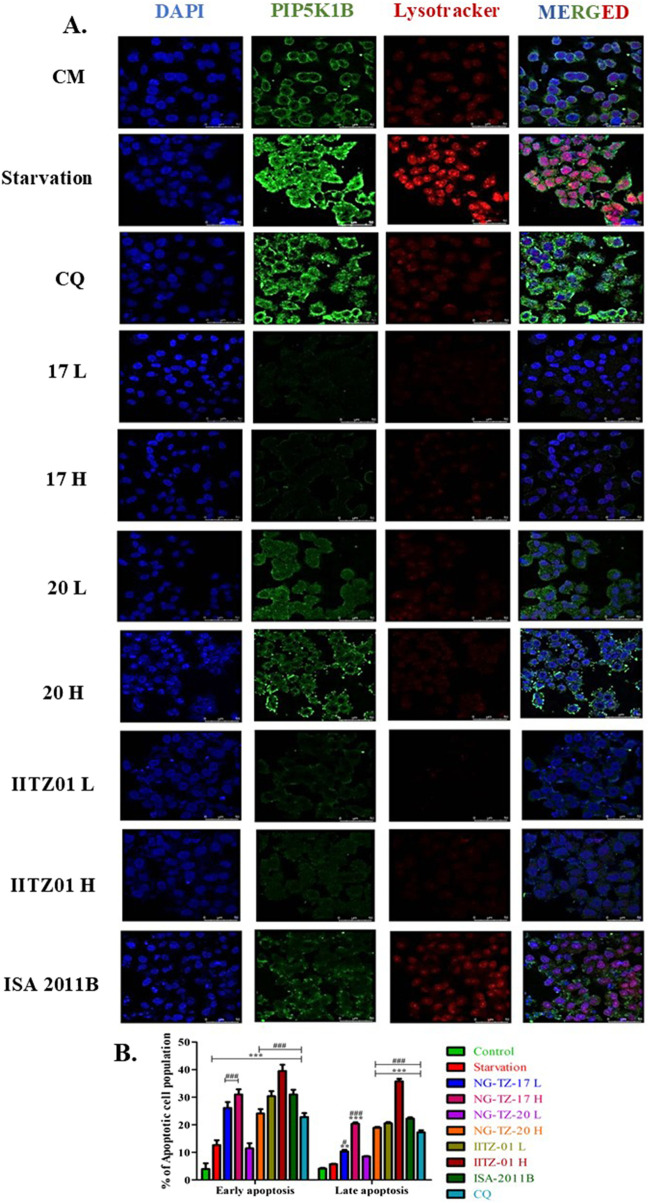
Treatment inhibited PIP5K1B and lysosome expression. **(A)** Confocal images of dual PIP5K and LysoTracker staining **(B)** Annexin V-PI assay. HepG2 cells were treated with IC_25_ (L) and IC_50_ (H) concentration of triazine compounds NG-TZ-17, IITZ01, NG-TZ-20, standard chloroquine (CQ), and ISA-2011B for 24 h, followed by dual PIP5K and LysoTracker staining and the Annexin V–PI assay. Treatment with PIP5K inhibitors decreased PIP5K1B and lysosome expression, indicating the inhibition of autophagy due to PIP5K1B following PIP5K immunofluorescence and LysoTracker dual staining. All images were captured using a confocal microscope, with ×63 magnification and 50 µm scale bar. The treatment with PIP5K also induced apoptosis, as indicated by an increase in early and late apoptotic cells in the Annexin V–PI assay. Statistical significance is indicated as follows: ** and *** denote *p* < 0.01 and *p* < 0.001, respectively, compared with the complete media control; #, ##, and ### denote *p* < 0.05, *p* < 0.01, and *p* < 0.001, respectively, compared with the serum starvation control. Data are presented as the mean ± SEM (n = 3) and analyzed using one-way ANOVA, followed by Tukey’s *post-hoc* test.

Overall, we observed that triazine PIP5K1B inhibitors NG-TZ-17 and IITZ01 showed better inhibition of proliferation than standard ISA-2011B (PIP5K1A inhibitor) and chloroquine (autophagy inhibitor). NG-TZ-20 proliferation inhibitory activity was equipotent to standard ISA-2011B and was superior to CQ as it showed at least a three-fold difference across all tested cell lines (Figure 3C). To explore the effect of triazine PIP5K inhibitors on autophagy, we treated HepG2 cells with two doses of NG-TZ-17 [0.5 μM (17L) and 1 μM (17H)], IITZ01 [0.5 μM (IITZ01 L) and 1 μM (IITZ01 H)], and NG-TZ-20 [2.83 μM (20L) and 5.66 μM (20H)]. Acridine orange-red staining results indicate that NG-TZ-17, IITZ01, and NG-TZ-20 deacidified acidic vesicles, resulting in autophagy inhibition. The treatment resulted in a decrease in the acidic vesicle of NG-TZ-17 (##, *p* < 0.01 and ###, 0.001 for NG-TZ-17 low and high concentrations, respectively, vs. starvation control); both concentrations of NG-TZ-20 and IITZ01 significantly reduced acidic vesicles (###, *p* < 0.001 vs. starvation control) (Figure 3D). To pinpoint the stage-specific inhibition of autophagy, we transfected HepG2 cells with the LC3B-GFP-RFP peptide, which was incorporated into autophagosomes when autophagy was stimulated. Yellow puncta were formed when acid-sensitive GFP-LC3B was not quenched in the acidic environment of autolysosomes, following lysosomal fusion. This occurs due to the merged fluorescence of acid-sensitive LC3B-GFP and acid-insensitive LC3B-RFP. Hence, to pinpoint the stage at which autophagy was inhibited, the ratio of yellow/red puncta was plotted. Treatment with the triazine PIP5K inhibitors NG-TZ-17 and NG-TZ-20 resulted in an increase in the yellow/red puncta ratio due to the accumulation of autophagosomes, indicating inhibition of autophagy at the autolysosomal step (Figures 3E,F). We explored the effect of NG-TZ-17 treatment on HepG2 PI-4,5-P_2_ levels. Serum starvation resulted in an increase in 4,5-P_2_ levels. These serum-starved cells were then treated with NG-TZ-17. The treatment resulted in depletion of PI-4,5-P_2_ levels (Figures 4A,B). The IC_50_ values of NG-TZ-17 was found to be 188 nM in inhibiting PI-4,5-P_2_ expression (Figure 4C). Furthermore, we performed PIP5K1B immunostaining and LysoTracker dual staining (Figure 5A). We observed that treatment with PIP5K inhibitors resulted in a decrease in PIP5K1B expression and lysosome turnover at high doses of NG-TZ-17, IITZ01, IITZ01, and ISA-2011B. The standard CQ successfully decreased the lysosomal turnover without affecting PIP5K1B expression (Figure 5A). Treatment with the PIP5K inhibitor also induced apoptosis, as indicated by an increase in early and late apoptotic cells in the Annexin V–PI assay (Figure 5B). Together, these findings demonstrate that triazine-based PIP5K1B inhibition effectively suppresses hepatic cancer cell survival by disrupting autophagy and triggering apoptotic pathways.

### 3.5 PIP5K inhibitors exacerbate ROS accumulation while suppressing PIP5K, autophagy, and Nrf2 signaling in H_2_O_2_-stimulated HepG2 cells

Tumor cells modulate ROS to maintain high proliferation (Liou and Storz, 2010). The tumor cells modulate the ROS switch between proliferation and adaptive pathways in response (Liou and Storz, 2010). Thus, to mimic the tumor microenvironment with elevated ROS, we stimulated HepG2 cells with a mild concentration of H_2_O_2_, i.e. 12.5 μM. The H_2_O_2_-exposed cells were then treated with two doses of NG-TZ-17 [(0.4 (17L) and 0.9 μM (17H)] and IITZ01 [0.5 (IITZ01 L) and 1 μM (IITZ01 H)], along with standards 7.76 μM of ISA-2011B (PIP5K1A inhibitor), 67.10 μM of chloroquine (autophagy inhibitor), and 5 μM ML-385 (Nrf2 inhibitor). We estimated the levels of mitochondrial superoxide, PIP5K1B, and lysosomal turnover, along with the expressions of PI3K/AKT, autophagy, and the Nrf2 antioxidant defense marker following treatment with inhibitors.

#### 3.5.1 PIP5K inhibitors aggravated H_2_O_2_-induced mitochondrial superoxides

**FIGURE 6:**
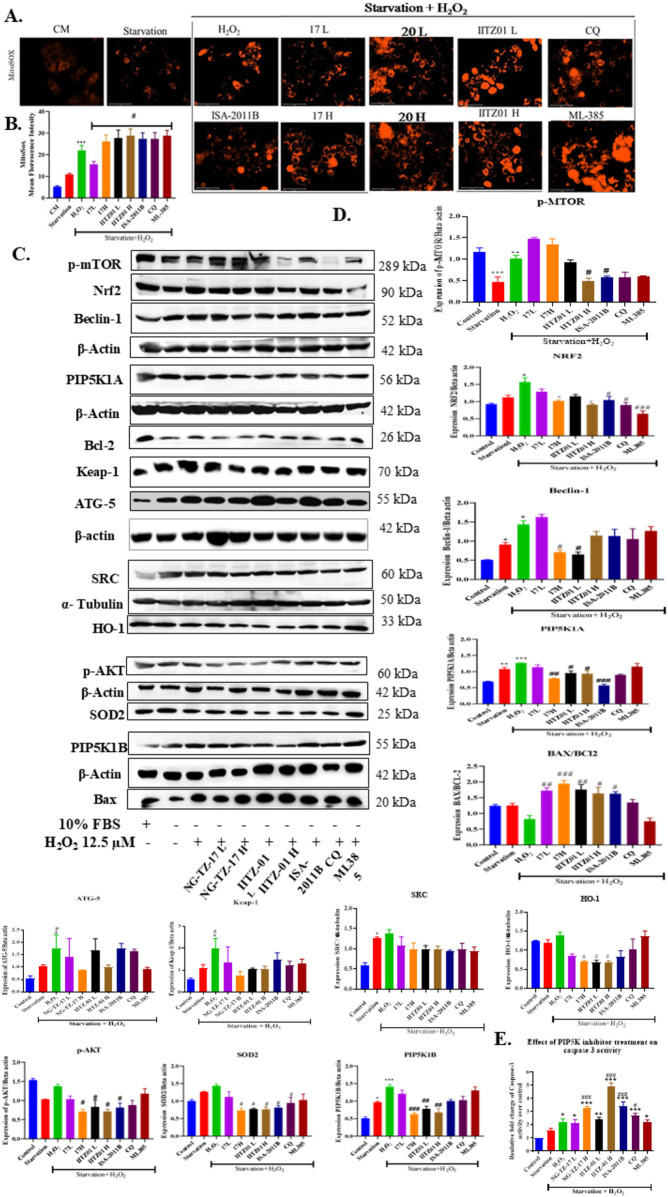
Treatment with PIP5K inhibitors sensitized HepG2 to a mild concentration of H_2_O_2_. **(A)** fluorescent microscopy images of HepG2 cells stained with MitoSOX, **(B)** Qualification graph of MitoSOX **(C, D)** displays western blot protein bands for various markers, including p-mTOR, Nrf2, Beclin-1, and others, with corresponding molecular weights, along with quantitative bar graphs for each protein’s expression ratio. **(E)** Effect on Caspase 3 activity.

The MitoSOX staining results revealed that mitochondrial superoxide levels were increased when stimulated by 12.5 μM of H_2_O_2_ (*, *p* < 0.05 vs. CM). Treatment with PIP5K inhibitors NG-TZ-17, IITZ01, and ISA-2011B, along with autophagy (CQ) and Nrf2 (ML-385) inhibitors, increased mitochondrial superoxide (Figures 6A,B). The effects of treatment with PIP5K inhibitors were similar to those of Nrf2 (ML-385) and autophagy inhibitors (chloroquine).

#### 3.5.2 PIP5K inhibitors suppressed H_2_O_2_-induced molecular changes

Furthermore, to evaluate the molecular effects of PIP5K inhibitor treatment on H_2_O_2_-exposed cells, we explored the effects of treatment on the PI3K/AKT, autophagy, and Nrf2 pathways. H_2_O_2_ (12.5 μM) exposure increased **p-mTOR** (**, *p* < 0.01), Nrf2 (*, *p* < 0.05), **PIP5K1B** (***, *p* < 0.001), **PIP5K1A** (***, *p* < 0.001), Beclin-1 (*, *p* < 0.05), **ATG5** (*, *p* < 0.05), Keap-1 (*, *p* < 0.05), and **SRC** (*, *p* < 0.05) compared to cell growth in complete media (Figures 5C,D). Treatment resulted in decreases in **p-mTOR** (#, *p* < 0.05, for both concentrations of IITZ01 H and ISA-2011B), Nrf2 (#, *p* < 0.05 for 17 H, IITZ01 H, ISA-2011B, and CQ; ###, *p* < 0.001 for ML-385), **PIP5K1B** (###, *p* < 0.001 for 17H and ##, *p* < 0.01 for both concentrations of IITZ01), **PIP5K1A** (##, *p* < 0.01 for 17H, #, *p* < 0.05 for both concentrations of IITZ01, and ###, *p* < 0.001 for ISA-2011B), **p-AKT** (#, *p* < 0.05 for 17H, both concentrations of IITZ01, and ISA-2011B), HO-1 (#, *p* < 0.05 for 17H and both concentrations of IITZ01), SOD2 (#, *p* < 0.05 for 17H, both concentrations of IITZ01, ISA-2011B, and CQ), Beclin-1 (#, *p* < 0.5 for 17H and IITZ01 L), the Bax/BCL-2 ratio (##, *p* < 0.01 for 17L, ###, *p* < 0.001, for 17H, and #, *p* < 0.05 for IITZ01 H), and no significant changes were observed in SRC expression levels compared to the 12.5 μM H_2_O_2_-exposed group (Figures 6C,D).

#### 3.5.3 Inhibition of PIP5K and lysosomal turnover lead to apoptosis

**FIGURE 7:**
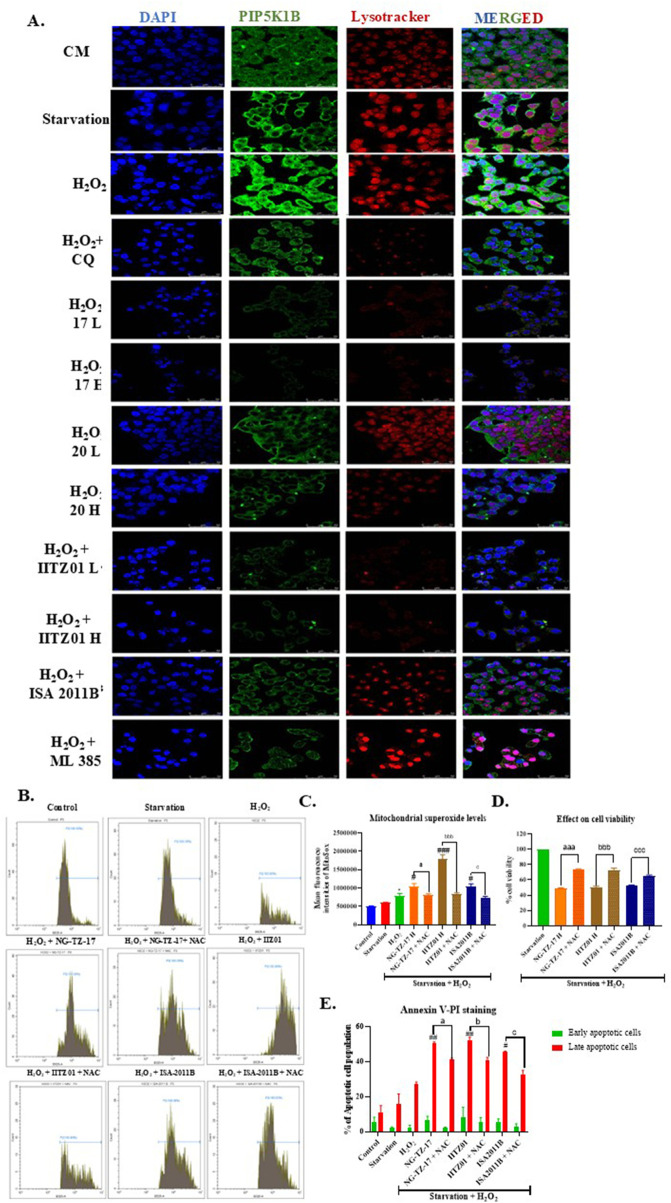
PIP5K inhibitors suppressed ROS-induced PIP5K1B and lysosomal expression, while free radical scavengers mitigated the effects of PIP5K inhibition. **(A)** PIP5K and LysoTracker dual staining **(B)** Histogram of the MitoSOX assay. **(C)** Quantification of the MitoSOX assay. **(D)** Effect of cellular viability. **(E)** Effect on apoptosis—Annexin V–PI assay. HepG2 cells were treated with IC_25_ (L) and IC_50_ (H) concentrations of triazine compounds (NG-TZ-17, IITZ01, and NG-TZ-20), standard CQ, and ISA-2011B, in the presence of 12.5 µM H_2_O_2_ for 24 h, followed by dual PIP5K and LysoTracker staining. Mild ROS induced PIP5K1B and lysosomal expression. CQ inhibited lysosomes only, and ML-385 had no effect; PIP5K inhibitors suppressed both. Confocal images were acquired at ×63 with a scale bar of 50 µm. In serum-starved HepG2 cells, H_2_O_2_ increased mitochondrial superoxide, which was further aggravated by PIP5K. Co-treatment with NAC reduced superoxide accumulation, improved cell viability, and decreased late apoptosis (Annexin V–PI assay). Data are presented as the mean ± SEM; statistical significance: *, *p* < 0.05; *, *p* < 0.001 vs. control; ##, *p* < 0.01; ###, *p* < 0.001 vs. H_2_O_2_; a, *p* < 0.05; aaa, *p* < 0.001 vs. H_2_O_2_ + NG-TZ-17 H; b, *p* < 0.05; bbb, *p* < 0.001 vs. H_2_O_2_ + IITZ01 H; c, *p* < 0.05; ccc, *p* < 0.001 vs. H_2_O_2_ + ISA-2011B H (n = 3).

Treatment with triazine PIP5K inhibitors resulted in a significant increase in caspase-3 activity and was superior to standards ISA-2011B, chloroquine, and ML-385. Furthermore, to validate the results of molecular analysis, we performed PIP5K1B and LysoTracker dual staining, where we observed that chloroquine and ML-385 did not affect PIP5K1B expression, although chloroquine inhibited autophagy, as indicated by an increase in LysoTracker fluorescence. Treatment with PIP5K inhibitors, including the investigational NG-TZ-17, NG-TZ-20, and IITZ01, along with standard ISA-2011B, resulted in a decrease in PIP5K1B and lysosomal turnover (Figure 7A).

ROS scavenging with N-acetylcysteine (NAC) rescues HCC cells from PIP5K-inhibitor-induced apoptosis:

Exposure of serum-starved HepG2 cells to H_2_O_2_ initially elevated mitochondrial superoxide levels (*p < 0.5), an effect that was further exacerbated upon treatment with PIP5K inhibitors NG-TZ-17 (#*p* < 0.5), IITZ01 (###*p* < 0.001), and ISA-2011B (#*p* < 0.5). Co-treatment with the antioxidant NAC effectively mitigated this response, significantly reducing superoxide accumulation and thereby improving cell viability (*p* < 0.001) (Figures 7B–D). Importantly, Annexin V–PI assays confirmed that NAC addition partially inhibited the progression of cells into the late apoptotic stage, which had been markedly induced by PIP5K inhibition (Figure 7E).

### 3.6 Inhibition of proliferative, autophagy, and Nrf2 pathways by PIP5K inhibitors reduced tumor burden in HepG2-induced hepatic cancer in SCID mice

**FIGURE 8:**
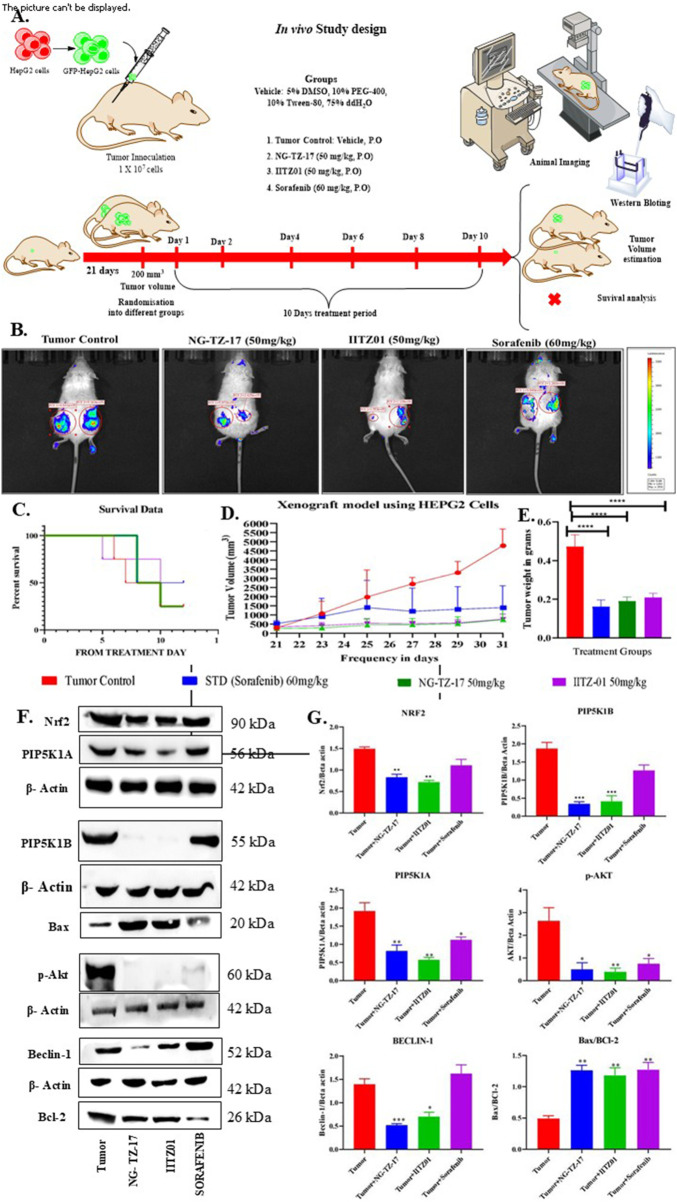
Treatment with PIP5K inhibitors reduced tumor burden in HepG2-induced hepatic cancer in SCID mice. Once tumors were established, HepG2–GFP tumor-bearing mice were divided into various groups: vehicle, NG-TZ-17, IITZ01, and sorafenib. NG-TZ-17 and IITZ01 were administered at 50 mg/kg, and standard sorafenib was administered orally at 60 mg/kg for 10 days. HepG2–GFP tumor images, volumes, weights, and survival data were recorded. **(A)** *In vivo study design.* Hepatic cancer was induced by subcutaneous injection of the GFP/HepG2 cell line. Once tumors were established, tumor-induced mice were treated with NG-TZ-17 (50 mg/kg), IITZ01 (50 mg/kg), and sorafenib (60 mg/kg) orally for 10 days daily. The treatment resulted in a decrease in tumor burden. The treatment shows that PIP5K inhibitors (NG-TZ-17 and IITZ01) were equipotent to standard sorafenib treatment. **(B)** *In vivo* imaging showing tumor burden. **(C)** Survival graph. **(D)** Tumor volume. **(E)** Tumor Weight. *, **, and *** represent *p* < 0.05, *p* < 0.01, and *p* < 0.001, respectively, when compared with the tumor control. Data are represented as the mean ± SEM, followed by one-way ANOVA with Tukey’s *post-hoc* test (n = 4). Further molecular expression analysis of isolated hepatic cancer tumors using Western blot exhibited a decrease in PIP5K1A, PIP5K1B, AKT, Beclin-1, and Nrf2 expression when treated with PIP5K inhibitors, whereas standard sorafenib treatment only inhibited AKT, with a minimal effect on PIP5K isoforms, Beclin-1, and Nrf2. However, treatment with both PIP5K inhibitors and sorafenib resulted in apoptosis activation, as indicated by the increase in the Bax/BCL-2 ratio. **(F)** Western blots. **(G)** Quantification of immunoblots. *, **, and *** represent *p* < 0.05, *p* < 0.01, and *p* < 0.001, respectively, when compared with the tumor control. Data are represented as the mean ± SEM, followed by one-way ANOVA with Tukey’s *post-hoc* test (n = 3).

GFP-tagged HepG2 cells were injected subcutaneously into the flank region of SCID mice. After the tumors attained a volume of 200 mm^3^ (Figure 8A), tumor-induced mice (n = 4/group) were treated with daily NG-TZ-17 (50 mg/kg), IITZ01 (50 mg/kg), and sorafenib (60 mg/kg) orally for 10 days. The treatment resulted in a decrease in tumor burden, tumor weight, and tumor volume (Figures 8B–E). The reduction in tumor burden was equipotent to sorafenib (***, *p* < 0.001 for NG-TZ-17, IITZ01, and sorafenib compared to tumor-bearing mice) (Figure 8E). The treatment with PIP5K inhibitors and sorafenib increased the overall survivability of the tumor-induced mice compared to control tumors (Figure 8C). Protein expression analysis of isolated hepatic cancer tumors was conducted using Western blot, which exhibited that treatment with PIP5K inhibitors (NG-TZ-17 and IITZ01) inhibited the proliferative pathway, as indicated by a decrease in PIP5K isoforms such as **PIP5K1A** (**, *p* < 0.01 for NG-TZ-17 and IITZ01 and *p < 0.05 for sorafenib), **PIP5K1B** (***, *p* < 0.001 for NG-TZ-17 and IITZ01), and **p-AKT** (*, *p* < 0.05 for NG-TZ-17 and sorafenib and **, *p* < 0.01 for IITZ01). PIP5K treatment also inhibited autophagy, as indicated by a reduction in the Beclin-1 (***, *p* < 0.001 for NG-TZ-17 and *, *p* < 0.05 for IITZ01) level. Inhibition of proliferative and autophagy pathways resulted in inhibition of the Nrf2-dependent antioxidant pathway, as indicated by depleting Nrf2 expression (**, *p* < 0.01 for NG-TZ-17 and IITZ01). Treatment with both PIP5K inhibitors (NG-TZ-17 and IITZ01) and sorafenib induced apoptosis, as indicated by an increase in the **Bax/BCL-2 ratio** expression (**, *p* < 0.01 for NG-TZ-17, IITZ01, and sorafenib), compared to the tumor-bearing group (Figures 8F,G).

## 4 Discussion

HCC is challenging to treat because malignant hepatocytes are embedded within normal hepatocytes with high metabolic capacity, leading to rapid drug metabolism and clearance. Many recently approved and investigational chemotherapeutic agents primarily target proliferative signaling pathways. However, blocking these pathways often induces cytoprotective autophagy, which contributes to therapy resistance and eventual treatment failure. For instance, the limited clinical success of PI3K/AKT/mTOR inhibitors in solid tumors has been partly attributed to compensatory activation of autophagy (Smith and Macleod, 2019). Moreover, inhibiting autophagy alone generally produces only cytostatic effects and may further drive resistance by activating the Nrf2-mediated antioxidant response pathway. Consequently, durable therapeutic responses are unlikely to result from targeting either proliferative or adaptive pathways in isolation. Growing evidence indicates that effective cancer treatment requires simultaneous suppression of both proliferative signaling and adaptive survival mechanisms (Sharma et al., 2014; Shantanu et al., 2021; Su et al., 2021; Mohsen et al., 2022). Accordingly, novel strategies that co-target these pathways may offer a more effective therapeutic approach for HCC and other solid tumors.

PI4,5P_2_, a secondary messenger produced by PIP5K, functions both as a substrate for PI3K signaling and as a recruiter of clathrin to facilitate lysosome recycling from autolysosomes during late autophagic events. PIP5K also plays a critical role in vesicular transport, membrane remodeling, ion channel regulation, cytoskeletal organization, and phagocytosis. PI4,5P_2_ also potentially modulates transcriptional processes by regulating the pre-mRNA splicing machinery and nuclear speckle localization (Jin and Xue, 2023). In mammals, six genes encode for kinases that are involved in the generation of PI4,5P_2_. PIP5K1A, PIP5K1B, and PIP5K1C generate PI4,5P_2_ from phosphatidylinositol-4-phosphate (PI4P), and PIP4K2A, PIP4K2B, and PIP42C generate PI4,5P_2_ from phosphatidylinositol-5-phosphate (PI5P). The availability of PI4P is >100 times higher than that of PI5P. This marked difference in substrate availability supports the hypothesis that PIP5K isoforms are extensively integrated into cell signaling pathways involved in tumor initiation and progression (Andrews et al., 2022). Thus, targeting PIP5K elicits a multifaceted response by inhibiting cancer proliferation and adaptation. PIP5K1A and PIP5K1B isoforms mediate PI3K/AKT and autophagy pathways, whereas PIP5K1C controls Rho/Rac1 signaling for cytoskeletal remodeling and cell motility (Burke, 2018).

Singhal et al. (1994) found that PIP5K is essential for hepatic cancer progression and can be an attractive target for its treatment (Singhal et al., 1994; Van den Bout and Divecha, 2009). Later, Peng et al. (2021) reported that upregulated PIP5K also contributed to increased tumor aggressiveness, invasion, and metastasis by manipulating filamin and cell adhesion proteins in patients with hepatocellular carcinoma. Furthermore, Semenas et al. (2014), Sarwar et al. (2019), and Peng et al. (2021) demonstrated a positive correlation between PIP5K1A and PIP5K1C expressions and the progression of prostate and hepatocellular carcinoma (Semenas et al., 2014; Peng et al., 2021). To the best of our knowledge, no reports are available on the role of PIP5K1B in hepatic cancer progression. Understanding the relevance of PIP5K in anticancer treatment, Semenas et al. (2014) and Martuza Sarwar identified ISA-2011B as a small-molecule PIP5K1A inhibitor and explored its efficacy for the treatment of prostate and breast cancer via PI3K/AKT inhibition (Semenas et al., 2014; Sarwar et al., 2019; Peng et al., 2021).

Several investigations have demonstrated that ROS regulates lipid kinases to alter adaptive (autophagy) and proliferative (PI3K/AKT) pathways, which, in turn, impact cellular growth, stasis, and death (Kim et al., 2018; George and Abrahamse, 2020). Research has shown that apoptotic stressors, such as peroxides and UV radiation, deplete PI4,5P_2_, which, in turn, leads to apoptotic cell death. However, overexpression of murine PIP5K1A (m-PIP5K1A, homologous to human PIP5K1B) prevented oxidative stress-induced PI4,5P_2_ depletion and apoptosis. This study also indicated that Src activation results in PIP5K phosphorylation and translocation away from the plasma membrane, where it activates survival signals mediated by ERK pathways (Gough, 2006). However, studies have not investigated how the modulation of PIP5K levels enables cells to counteract increased ROS levels that drive liver cancer growth and resistance. The mechanism by which PIP5K modulation with a small-molecule inhibitor affects downstream signaling pathways such as PI3K/AKT, autophagy, and Nrf2 in response to ROS remains unknown. Since PIP5K1A and PIP5K1B are key isoforms in PI3K/AKT and autophagy signaling, this study used isoform-specific PIP5K inhibitors to determine their role in ROS-assisted HCC progression.

In this study, we estimated PIP5K isoforms (PIP5K1A and PIP5K1B), autophagy (Beclin-1), and Nrf2 expression in 36 progressive grades of hepatic cancer patients and compared them with normal adjacent controls. We observed that the expressions of PIP5K isoforms, Beclin-1, and Nrf2 increased as the grade progressed, indicating a positive correlation between the markers (Figures 1A–D). Our results matched those of Semenas et al. (2014) and Peng et al. (2021), who observed an increase in PIP5K1A and PIP5K1C levels as prostate cancer and HCC progressed. Our results also correlated with studies linking Beclin-1 and Nrf2 levels to hepatocellular carcinoma progression (Liang et al., 2018; Lee et al., 2020; Ehsan et al., 2021). These results reveal a link between PIP5K, autophagy, and antioxidant defense pathways that warrants further experimentation and validation before becoming a target. The basal expression levels of PIP5K isoforms, autophagy markers, and Nrf2 in hepatic cancer cell lines (PRF5, SNU-387, SK-Hep-1, and HepG2) showed a similar pattern. Accumulating scientific evidence has shown that elevated PIP5K isoforms increase autophagy and Nrf2-mediated antioxidant enzymes, consistent with the reported data. Among the panel of cells, HepG2 cells showed the highest expression levels of PIP5K isoforms, autophagy, and Nrf2-mediated antioxidant markers (Figures 1E,F). Thus, HepG2 was chosen as the cell line for further studies. To investigate the effect of oxidative stress on hepatic cancer cells, we exposed HepG2 cells to an increasing concentration of H_2_O_2_. We measured the resulting reactive oxygen species levels, autophagy flux, and makers of proliferation, autophagy, and Nrf2 (Figure 2A). We observed that serum starvation, along with lower H_2_O_2_ concentrations (≤12.5 µM), promoted proliferation, whereas moderate H_2_O_2_ levels (25 and 50 µM) induced a state of stasis, in which neither proliferation nor death occurred. However, at H_2_O_2_ concentrations >100 μM, HepG2 cells showed a significant loss of cell viability (Ohguro et al., 1999). In contrast, HepG2 cells exposed to increasing concentrations of H_2_O_2_ in complete media did not exhibit this trend. This explains why cells maintain a higher proliferative state even under scarce nutrient supply (Yamashita and Wang, 2013; Nazio et al., 2019). The mitochondrial superoxide and autophagy markers were correspondingly increased with H_2_O_2_ concentration, with mitochondrial superoxides and autophagy flux highest at cytotoxic doses, as indicated by MitoSOX and LysoTracker staining, respectively. High levels of ROS generated mitochondrial superoxides, thereby leading to autophagic cell death (Figures 2B–E). To understand the relationship between PIP5K isoforms and proliferation, autophagy, and antioxidant defense mechanisms as ROS levels increased, we measured the levels of PIP5K1A, PIP5K1B, SRC, Beclin-1, Nrf2, SOD2, HO-1, Bax, and BCL-2. We observed that as ROS increased, it induced expression of PIP5K isoforms, Beclin-1, HO-1, and SOD2 expressions, without a significant increase in Nrf2 or the Bax/BCL-2 ratio in HepG2 cells. This suggests that increased levels of proliferative and autophagy proteins promoted antioxidant enzyme levels by allowing Nrf2 to translocate to the nucleus. Elevated ROS reduces Keap-1’s ability to form an inhibitory complex with Nrf2 and is degraded by autophagy, thereby facilitating Nrf2 nuclear translocation and the expression of antioxidant genes such as HO-1 and SOD2 (Figures 2E,F) (Jiang et al., 2015; Koundouros and Poulogiannis, 2018). However, at cytotoxic levels, as shown in previous studies, the expression of PIP5K isoforms and proliferative markers decreases as the SRC kinase-mediated feedback pathway is activated, which indicates the shift from a proliferative state to stasis and death by maintaining elevated autophagy levels (Halstead et al., 2006; Chen et al., 2009; Hou et al., 2019). The sustained activation of autophagy leads to apoptosis, as indicated by an increase in the Bax/BCL-2 ratio and elevated caspase 3 levels, which are highest at cytotoxic concentrations of 100 and 200 µM. Our findings are consistent with the reports of Halstead et al. (2006) and Gough (2006), demonstrating that overexpression of PIP5K confers protection against hydrogen peroxide-induced oxidative stress. Elevated hydrogen peroxide concentrations inhibit PIP5K activity and deplete cellular PI4,5P_2_ levels, thereby promoting apoptosis. Mechanistically, hydrogen peroxide triggers tyrosine phosphorylation and induces the translocation of PIP5K away from the plasma membrane, which depend on SRC kinase activity. Importantly, overexpression of PIP5K counteracted hydrogen peroxide-mediated cytotoxicity by activating the ERK signaling pathway, whereas pharmacological inhibition of ERK re-sensitized PIP5K-overexpressing cells to hydrogen peroxide exposure (Halstead et al., 2006). This suggests that available PIP5K isoforms are used to maintain elevated autophagy levels, which, in turn, lead to autophagic cell death by activating apoptosis (Halstead et al., 2006; Dall'Armi et al., 2013; Tang et al., 2002).

We identified that IITZ01 (an autophagy inhibitor) and its derivatives inhibited PIP5K in cell-free kinase assays (Figure 3A). IC_50_ values for the identified inhibitors, NG-TZ-17, IITZ01, and NG-TZ-20, were 239, 281, and 691 nM, respectively, against the PIP5K1B enzyme (Figure 3B). PIP5K inhibitors were superior at inhibiting cellular proliferation in HCC cell lines compared to standards ISA-2011B and chloroquine (Figure 3C). The PIP5K inhibitor retained autophagy inhibition, as characterized by acridine-orange staining, GFP-RFP-LC3B baculovirus transfection, and dual PIP5K1B and LysoTracker staining. The reduction in acridine orange and red (650 nm) fluorescence, PI4,5P_2_, PIP5K1B, and lysosomes, along with an increase in yellow/red puncta, indicates inhibition of autophagy flux following treatment with PIP5K inhibitors (Figures 3D–F, 4). The IC_50_ value of NG-TZ-17 for PI4,5P2 depletion in HepG2 cells was found to be 188 ± 33 nM (Figure 4C). Treatment with triazine inhibitors inhibited PIP5K1B and lysosomal activity, as demonstrated through dual staining with PIP5K1B and LysoTracker (Figure 5A). This dual inhibition suggests suppression of both autophagy and proliferative signaling pathways, leading to an increased population of cells undergoing early- and late-stage apoptosis, as shown using the Annexin V–PI staining assay (Figure 5B).

Since ROS modulated PIP5K expression and cellular proliferation, treating them with pharmacological inhibitors of PIP5K at a proliferative concentration of H_2_O_2_ should sensitize cells to apoptosis. Thus, we exposed cells to mild ROS (12.5 μM H_2_O_2_) following serum starvation, treated cells with different concentrations of PIP5K inhibitors such as NG-TZ-17 and IITZ01, and compared the results with standard PIP5K1A (ISA-2011B), autophagy (CQ), and Nrf2 (ML385) inhibitors. We observed that PIP5K inhibitors increased intracellular mitochondrial superoxides (Figures 6A,B) and were able to inhibit both proliferative PI3K/AKT and autophagy pathways, which decreased the expression of Nrf2 and other Nrf2-mediated antioxidant genes compared to standard PIP5K (ISA-2011B), autophagy (CQ), and Nrf2 (ML385) inhibitors (Figures 6C,D). Treatment with PIP5K inhibitors significantly increased caspase-3 activity, thereby indicating the induction of cellular apoptosis. Triazine inhibitors were more potent and effective in increasing caspase-3 activity than the standard agents ISA-2011B, chloroquine, and ML-385 (Figure 6E). Further investigation revealed that apoptosis was primarily driven by the dual inhibition of PIP5K1B and lysosomal turnover (Figure 7A). To validate our experimental findings, we treated HCC cells exposed to H_2_O_2_ with PIP5K inhibitors, both in the presence and absence of the antioxidant NAC. We then estimated mitochondrial superoxide levels, cell viability, and apoptotic responses. H_2_O_2_ exposure increased mitochondrial superoxide levels, which were further exacerbated by PIP5K inhibition. This was accompanied by a marked reduction in cellular viability and an increase in late apoptotic populations (Figures 7B–D). Notably, co-treatment with NAC attenuated these effects, mitigating superoxide accumulation, loss of viability, and apoptosis. The addition of NAC partially counteracted the effects mediated by PIP5K inhibition, which is consistent with our hypothesis that ROS sensitization represents a key mechanism underlying the cytotoxicity of PIP5K inhibitors. By attenuating oxidative stress, NAC reduced mitochondrial superoxide accumulation, thereby delaying cell progression into late apoptosis (Figure 7D). Collectively, these results indicate that PIP5K inhibition sensitizes HCC cells to ROS-mediated cytotoxicity.

This dual blockade likely disrupted proliferative (PI3K/AKT) and adaptive (autophagy) survival mechanisms, culminating in the depletion of Nrf2 and its downstream antioxidant defenses, thereby sensitizing HepG2 cells to apoptosis under proliferative ROS conditions. Notably, PIP5K inhibitors demonstrated superior potency across these endpoints. The PIP5K inhibitors were more potent and overcame the limitations of individual autophagy (CQ) and Nrf2 (ML385) inhibitors.

Treatment with PIP5K inhibitors NG-TZ-17 and IITZ01 at 50 mg/kg orally, administered daily for 10 days, reduced tumor burden and tumor mass and improved overall survival of hepatic cancer-induced SCID mice. The overall *in vivo* efficacy of PIP5K inhibitors was similar to that of standard sorafenib administered orally at 60 mg/kg daily for 10 days (Figures 8B–E). Results from the molecular analysis of isolated tumors revealed that treatment with PIP5K inhibitors (NG-TZ-17 and IITZ01) and sorafenib enhanced apoptosis in cancer cells, as evidenced by an increase in the Bax/BCL-2 ratio. The treatment with PIP5K inhibitors resulted in dual inhibition of proliferative and adaptive (autophagy and Nrf2) pathways, as indicated by a decrease in PIP5K isoforms (PIP5K1A and PIP5K1B), AKT, Beclin-1, and Nrf2, compared to only PIP5K1A and AKT inhibition in the case of sorafenib treatment (Figures 8F,G). PIP5K inhibitor treatment offers a dual targeting mechanism that addresses a critical limitation of sorafenib: its failure to suppress autophagy and Nrf2 during long-term therapy. Gao et al. (2021) demonstrated that Nrf2 signaling promotes cancer stemness and chemoresistance in sorafenib-treated HCC cells (Gao et al., 2021). These results demonstrate the potential advantages of using PIP5K inhibitors over sorafenib for HCC treatment as they inhibit both proliferative and adaptive pathways. Further studies are required to examine the effects of PIP5K inhibitors on ROS-mediated cellular apoptosis in sorafenib-induced hepatic cancer resistance.

Overall, we demonstrate that PIP5K plays a critical role in mediating cellular proliferation and adaptive responses against excessive ROS. Inhibition of PIP5K appears to be critical for sensitizing cancer cells to even low levels of ROS. Under normal conditions, such ROS levels promote proliferation in the absence of PIP5K inhibitors. However, when PIP5K is blocked, the same oxidative stress compromises the survival capacity of cancer cells. Although both PIP5K1A and PIP5K1B are involved in PI3K–AKT and autophagy signaling pathways, *in vitro* findings indicate that IITZ01 and NG-TZ-17 more effectively inhibited proliferative (PI3K–AKT) and autophagy pathways compared with the standard PIP5K1A inhibitor ISA-2011B. This enhanced efficacy may be attributed to improved cellular permeability and effective lysosomotropic properties in cancer cells. Further Caco-2 permeability and pharmacokinetic (PK) studies are necessary to determine the precise mechanisms underlying their superior activity.

We also examined the role of PIP5K in HCC progression and identified NG-TZ-17 and IITZ01 as potential PIP5K1B inhibitors with anticancer activity against HCC. However, these findings remain preliminary due to the limited sample size, including 36 human HCC specimens and an *in vivo* cohort of four mice per group in the HepG2 cell-induced tumor model in SCID mice. Validation in larger and more diverse cohorts is required to establish the robustness, reproducibility, and translational relevance of IITZ01 and NG-TZ-17 for hepatic cancer treatment.

Although NG-TZ-17 demonstrated more potent inhibition of PIP5K1B compared with IITZ01, further studies are needed to evaluate its potential off-target effects across a broad kinase panel. Additional mechanistic investigations, such as measuring ROS levels in transplanted tissues and assessing the impact of PIP5K inhibition in PIP5K-overexpressing cells, would further elucidate the regulation of the PIP5K–autophagy–Nrf2 axis.

Finally, comprehensive pharmacological characterization, including detailed pharmacokinetic profiling, bioavailability assessment, and systemic toxicity evaluation, is essential before considering the clinical translation of these triazine-derived PIP5K inhibitors (IITZ01 and NG-TZ-17).

### 4.1 Conclusion

The study highlights how PIP5K plays a crucial role in cancer proliferation and adaptation by modulating ROS through PIP5K–autophagy–Nrf2 signaling. The modulation of ROS enables cancer cells to maintain a proliferative state even under nutrient-scarce conditions and to tide over adverse conditions. Pharmacological inhibition by PIP5K with NG-TZ-17, IITZ01, and ISA-2011B sensitized HCC cells to mild ROS levels *in vitro*. Treatment with the PIP5K inhibitors NG-TZ-17 and IITZ01 reduced tumor burden in HCC-induced SCID mice and was equipotent to standard sorafenib *in vivo*. PIP5K inhibitors overcome the limitations of standard sorafenib treatment by exhibiting dual inhibition of the PI3K/AKT and autophagy pathways.

## Data Availability

The original contributions presented in the study are included in the article/supplementary material; further inquiries can be directed to the corresponding authors.
